# Cost of illness for childhood diarrhea in low- and middle-income countries: a systematic review of evidence and modelled estimates

**DOI:** 10.1186/s12889-020-08595-8

**Published:** 2020-05-05

**Authors:** Ranju Baral, Justice Nonvignon, Frédéric Debellut, Samuel Agyei Agyemang, Andrew Clark, Clint Pecenka

**Affiliations:** 1 PATH, Seattle, USA; 2 Department of Health Policy, Planning and Management, School of Public Health, University of Ghana, Legon, Ghana; 3 Health Economics, Systems and Policy Research Group, University of Ghana, Legon, Accra, Ghana; 4 PATH, Geneva, Switzerland; 5 London School of Hygiene and Tropical Medicine, London, UK

**Keywords:** Cost of illness, Cost, Diarrhea

## Abstract

**Background:**

Numerous studies have reported the economic burden of childhood diarrhea in low- and middle-income countries (LMICs). Yet, empirical data on the cost of diarrheal illness is sparse, particularly in LMICs. In this study we review the existing literature on the cost of childhood diarrhea in LMICs and generate comparable estimates of cost of diarrhea across 137 LMICs.

**Methods:**

The systematic literature review included all articles reporting cost estimates of diarrhea illness and treatment from LMICs published between January 2006 and July 2018. To generate country-specific costs, we used service delivery unit costs from the World Health Organization’s Choosing Interventions that are Cost-Effective (WHO–CHOICE database). Non-medical costs were calculated using the ratio between direct medical and direct non-medical costs, derived from the literature review. Indirect costs (lost wages to caregivers) were calculated by multiplying the average GDP per capita per day by the average number of days lost to illness identified from the literature. All cost estimates are reported in 2015 USD. We also generated estimates using the IHME’s service delivery unit costs to explore input sensitivity on modelled cost estimates.

**Results:**

We identified 25 articles with 64 data points on either direct or indirect cost of diarrhoeal illness in children aged < 5 years in 20 LMICs. Of the 64 data points, 17 were on the cost of outpatient care, 28 were on the cost of inpatient care, and 19 were unspecified. The average cost of illness was US$36.56 (median $15.73; range $4.30 – $145.47) per outpatient episode and $159.90 (median $85.85; range $41.01 – $538.33) per inpatient episode. Direct medical costs accounted for 79% (83% for inpatient and 74% for outpatient) of the total direct costs. Our modelled estimates, across all 137 countries, averaged (weighted) $52.16 (median $47.56; range $8.81 – $201.91) per outpatient episode and $216.36 (median $177.20; range $23.77 –$1225.36) per inpatient episode. In the 12 countries with primary data, there was reasonable agreement between our modelled estimates and the reported data (Pearson’s correlation coefficient = .75).

**Conclusion:**

Our modelled estimates generally correspond to estimates observed in the literature, with a few exceptions. These estimates can serve as useful inputs for planning and prioritizing appropriate health interventions for childhood diarrheal diseases in LMICs in the absence of empirical data.

## Background

Diarrheal deaths in children have declined by more than 55% globally [1–3] since 2000. Despite impressive gains, diarrhea remains one of the leading causes of global disease burden, accounting for approximately 1.1 billion episodes, 450,000 deaths, and 40 million disability-adjusted life-years (DALYs), among children under five in 2016. Countries in South Asia and sub-Saharan Africa account for almost 90% of global diarrheal deaths in children and a significant share of the total disease burden [3].

Childhood diarrhea imposes economic costs on the health system and families. These costs are especially poignant in resource-limited settings. Often referred to as disease of poverty, repeated bouts of diarrhea can lead to malnutrition, stunting and delayed brain growth later in life costing individuals and societies substantial economic burden [4–6]. Proven solutions to cost -effectively address diarrhea are available, including interventions like oral rehydration therapy [7], micronutrient supplementation [8], rotavirus vaccines [9, 10], as well as general improvements in water and sanitation [11]. Increasing access to existing solutions will be important to prevent and further lower diarrheal disease burden [12].

Cost has been shown to hinder households’ access to diarrhea treatment in many countries [13]. Diarrheal disease largely contributes to the vicious cycle of ill health and poverty due to economic burden [14, 15]. Although studies have reported costs of treating diarrhea in a number of countries (e.g., Malawi [16], Colombia [17], Bolivia [18], Bangladesh [19]), diarrhea treatment costs are not universally known across low- and middle-income countries. A previous systematic review [20] included only studies focusing on Latin America and the Caribbean. As data on cost of illness is a useful input for prioritizing, selecting, and scaling up appropriate health interventions, country-specific estimates of the costs of diarrhea illness are important for policy and advocacy purposes.

The objective of this analysis was to provide more comprehensive diarrhea illness cost estimates in low-and middle-income countries. To achieve this objective, we first conducted a systematic review of empirical studies on cost of diarrhea illness and treatment among children under age five in low-and middle-income countries. We then generated modelled country-specific estimates of diarrhea treatment cost for 137 low-and middle-income countries. We also compared our modelled estimates to the empirical literature, when available, as a consistency check in our efforts to extend diarrhea cost estimates beyond the countries that have empirical estimates available. The cost estimates generated are intended to be useful inputs to estimating the costs of managing diarrheal diseases both globally and, particularly, in settings where data are lacking.

## Methods

### Review of published studies

We conducted a systematic literature search to identify all published peer-reviewed studies reporting cost of diarrhea and rotavirus illness and treatment among children under 5 years. The search was limited to include studies from low-and middle-income countries published in English language between January 2006 and July 2018. Studies from high-income countries, and those that did not involve primary data collection and reported only modelled estimates of cost data were excluded.

**Fig. 1:**
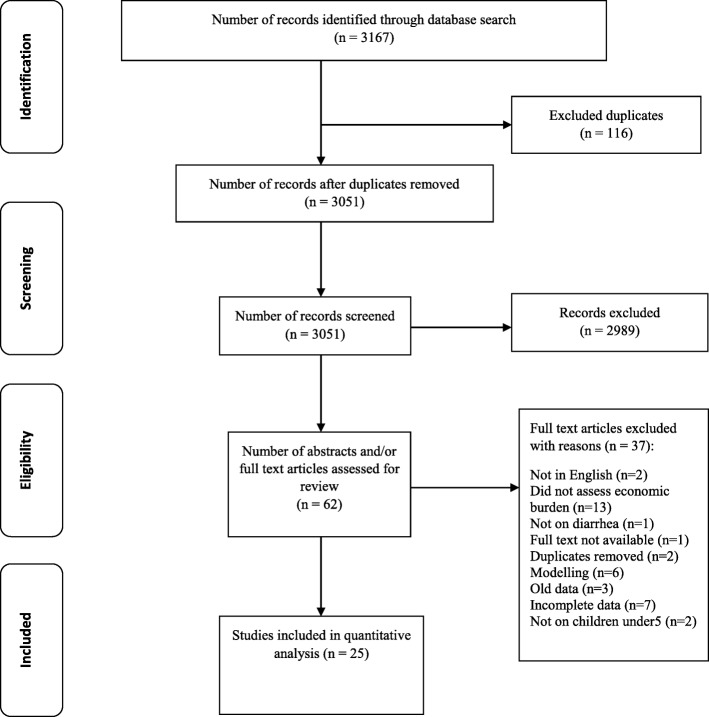
PRISMA flow diagram of the literature search

Literature searches were conducted in the following electronic databases: Embase Ovid Excerpta (PUBMED/MEDLINE), Cochrane Library, Web of Science core collections, EconLit, and Google Scholar using the key search terms “Direct healthcare cost”, “Indirect healthcare cost”, “Burden of illness”, “Diarrhea”, “Rotavirus”, “Low-income countries” and “Middle-income countries”. A full overview of the electronic search strategies used for different databases is provided in Additional file 1. Studies included for final review had to report the empirical costs of diarrhea illness. Data on direct medical costs, direct non-medical costs, and indirect costs of treating a diarrhea episode at any level of health care facilities, as well as the cost per episode of diarrhea, either for inpatient or outpatient care setting, were collected as primary study outcomes. The multiple database searches were stored in EndNote X7 (Thompson Reuters, CA, USA). A full review protocol was developed at the beginning of this study and is available from PROSPERO (PROSPERO 2018: CRD42019119816). We used the Preferred Reporting Items for Systematic Reviews and Meta-Analysis (PRISMA) guidelines as a guide in identifying relevant studies for the review. A PRISMA flow chart illustrating the selection process is shown in Fig. 1.

Three authors (SAA, RB, JN) independently screened all identified relevant articles based on the title and abstract. Any disagreements in study selection were resolved through discussion among authors (RB, CP, JN, SAA) after a review of the full-text of potentially relevant studies. All relevant studies thus selected were retrieved and reviewed for full text. Data extraction was performed independently by two authors (SAA, RB). Extracted data were discussed and discrepancies were resolved before final compilation of extracted data into a pre-developed Excel template. Extracted data from each study were aggregated and/or re-categorized where needed to obtain harmonized and comparable measures of primary outcomes; i.e., direct medical, direct non-medical, indirect, and total cost per inpatient and outpatient episode treated.

Cost estimates extracted from the included studies were converted and presented in 2015 USD units. For currency conversion to standard 2015 USD units, cost estimates in the source literature were first converted to local currency units (LCU) for the given year, then inflated to 2015 LCU using country-specific annual inflation rates, and then converted to 2015 USD [21]. GDP deflator values were used to adjust for inflation, and official exchanges rates were used to convert LCU to USD. Both data series were obtained from the World Bank’s World Development Indicators database [22].

### Comparison of cost estimates

To assess the within-country consistency of reported estimates, we examined estimates for countries with multiple costing studies and a comparable metric. We used direct medical costs for either inpatient or outpatient visits as the comparable metric, as they were a measurable and commonly reported component of total costs.

### Modelling diarrhea cost of illness

**Fig. 2:**
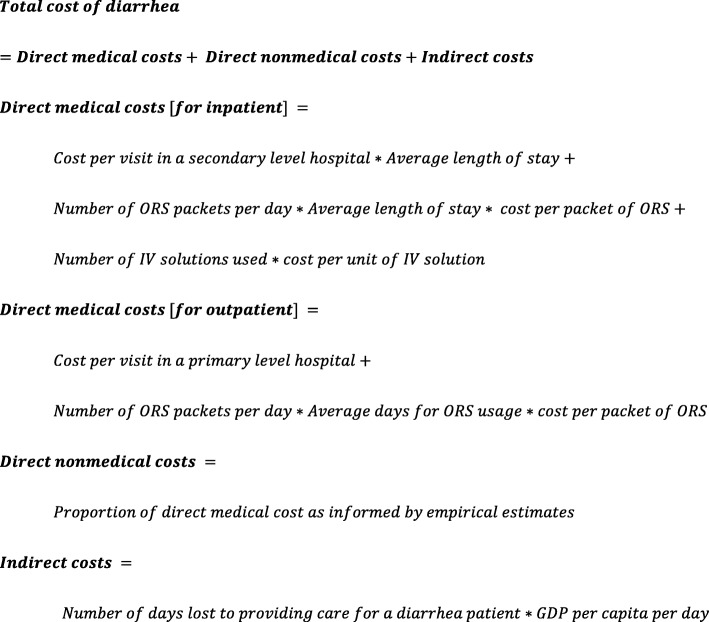
Methods for calculating comparable estimates of cost of diarrhea illness and treatment

To model diarrhea cost of illness in 137 low-and middle-income countries, we first estimated the direct medical costs, direct non-medical costs, and indirect costs. We then aggregated these three cost components to estimate total costs for both inpatient and outpatient episodes of diarrhea (see Fig. 2).

Direct medical cost estimates are based on the WHO-CHOICE database, which provides country-specific service delivery unit cost estimates [23], as well as costs of commodities typically used in diarrhea treatment. Using inflation-adjusted 2010 WHO-CHOICE unit cost estimates received from WHO, we converted them to 2015 USD using methods described above. For inpatient costs, we used country-specific bed day costs estimates at secondary level hospitals, assuming an intermediate level of care, and a four-day length of stay as used elsewhere [10]. Based on the recommendation from disease experts, we assumed that ORS would be given to any diarrhea case after each stool. We assume that one inpatient diarrhea case would receive six packs of oral rehydration solution (ORS) treatment per day in the facility as well as two intravenous (IV) solutions during their stay *(assumptions based on expert opinion)*. Per MSH’s International Medical Products Price Guide, we value each packet of ORS at $0.29 and IV solution at $0.55 [24]. Outpatient direct medical costs were estimated in a similar manner. The country-specific cost per outpatient visit was also taken from WHO-CHOICE using a primary care hospital as the treatment setting. Outpatient cases were assumed to receive six ORS packets per day for 2 days.

Direct non-medical costs build on direct medical cost calculations as well as empirical cost estimates from the literature. Most studies included in our systematic review only reported either the total direct cost and/or direct medical costs but not direct non-medical costs. We therefore calculated the direct medical cost share as a proportion of total direct costs in the papers in which these values were presented. We then averaged the direct medical cost share across studies and multiplied our modelled country-specific estimate of direct medical costs by the reciprocal of the average direct medical cost share, which resulted in total direct cost estimates for each country. After obtaining total direct costs, we calculated direct non-medical costs by multiplying total direct cost estimates with the direct non-medical cost share. Separate calculations were made for outpatient and inpatient illness episodes using respective cost shares from the literature.

Indirect costs estimates were sparse and inconsistently reported in the literature. To calculate indirect cost of illness, we used income lost to providing care for the sick children. We utilize 2015 World Bank’s gross domestic product (GDP) per capita estimates for each country and divided by 365 to obtain a daily estimate of the value of a lost caretaker day [25]. We assume inpatient caretakers lose 8.4 productive days and outpatient caretakers lose 4.3 productive days as based on the literature [26]. We then multiplied the number of days lost to providing care for sick children by the GDP per capita per day to estimate indirect costs.

As a sensitivity test to the primary inputs used for modelling country specific cost of illness, the WHO-CHOICE unit cost estimates, we also generated estimates using a more recently published IHME service delivery unit cost estimates [27] and compared the results generated using the two sources.

## Results

### Search results from review of published studies

We identified 3167 studies through our database search. Upon excluding duplicate records, we screened 3051 studies for relevance of which only 62 were eligible for full text review. Twenty-five of the studies met the inclusion criteria and were included in the final review and data extraction (Fig. 1).

### Characteristics of studies included

Studies identified for quantitative analysis in the systematic review (*N* = 25) represented six low- income countries (LIC) [16, 28–32], nine lower-middle-income countries (LMIC) [18, 19, 30, 33–44], and five upper-middle-income countries (UMIC) [13, 17, 45–47], as classified by the World Bank’s income groups in 2018 [25]. While most studies reported using societal perspective (22/25, 88%) in measuring cost [13, 17–19, 29–32, 34–37, 39, 41–49], we observed a wide variation in use of term societal perspective across those studies. One study each reported using payer [33] and provider [28] perspectives*.* One study [40] did not explicitly report the perspective used. Seven studies exclusively focused on measuring inpatient costs [13, 31, 34, 35, 38, 40, 46]. The remaining studies [18/25] included costs of both inpatient and outpatient care [16–19, 28–30, 32, 33, 36, 37, 39, 41–45, 47] although seven of those studies did not explicitly distinguish between costs incurred for either outpatient or inpatient care (see Table 1). There were 64 unique data points on either direct or indirect cost of diarrheal illness in children. Of these, 17 data points were on the cost of outpatient care, 28 on inpatient care, and the remaining 19 were not specific to outpatient or inpatient care. We generated and reported separate estimates from those studies.

**Table 1:** Characteristics of studies included in systematic review

Reference	Country (ies)	Income group (s)	Currency, year	Disease focus	Costing perspective	Sensitivity analysis	Study focus [Inpatient (IP) / Outpatient (OP)/ Both]
Ruhago et al., 2015 [28]	Tanzania	LIC	2012, USD	Diarrhea	Provider	Yes	Both
Rheingans et al., 2012 [B] [29]	India; Bangladesh; Pakistan	LIC	2011, USD	Diarrhea	Societal	No	Both but does not distinguish OP/IP
Ngabo et al., 2016 [31]	Rwanda	LIC	2014, USD	Diarrhea	Societal	No	IP
Memirie et al., 2017 [32]	Ethiopia	LIC	2013, USD	Diarrhea	Societal	No	Both
Hendrix et al., 2017 [16]	Malawi	LIC	2014, USD	Diarrhea	Societal	No	Both
Rheingans et al., 2012 [A] [30]	Gambia; Kenya; Mali	LMIC; LIC	2011, USD	Diarrhea	Societal	No	Both but does not distinguish OP/IP
Wilopo et al., 2009 [33]	Indonesia	LMIC	2007, USD	Rota	Payer	Yes	Both
Sowmyanarayanan et al., 2012 [34]	India	LMIC	2009, INR	Rota	Societal	No	IP
Soltani et al., 2015 [35]	Tunisia	LMIC	2015, TND	Rota	Societal	Yes	IP
Sarker et al., 2018 [36]	Bangladesh	LMIC	2014, USD	Diarrhea	Societal	Yes	Both
Riewpaiboon et al., 2016 [37]	Vietnam	LMIC	2014, USD	Rota	Societal	Yes	Both but does not distinguish OP/IP
Osano et al., 2011 [38]	Kenya	LMIC	2008, KNH	Rota	Societal	No	IP
Mendelsohn et al., 2008 [39]	India	LMIC	2006, USD	Diarrhea	Societal	No	Both
Mathew et al., 2016 [40]	India	LMIC	2014, INR	Rota	NR	No	IP
Jacob et al., 2016 [41]	India	LMIC	2014, INR	Rota	Societal	No	Both
Halder et al., 2017 [19]	Bangladesh	LMIC	2007, USD	Diarrhea	Societal	No	Both
Flem et al., 2009 [42]	Kyrgyzstan	LMIC	2008, USD	Rota	Societal	Yes	Both but OP is not used as it is modeled
Das et al., 2015 [43]	Bangladesh	LMIC	2011, USD	Rota	Societal	No	Both but does not distinguish OP/IP
Burke et al.,2014 [44]	Bolivia	LMIC	2011, USD	Diarrhea	Societal	No	Both
Burke et al.,2013 [18]	Bolivia	LMIC	2011, USD	Diarrhea	Societal	No	Both
Zhang et al., 2015 [45]	China	UMIC	2013, USD	Rota	Societal	No	Both
Phavichitr et al., 2013 [46]	Thailand	UMIC	2011, THB	Diarrhea	Societal	No	IP
Latipov et al., 2011 [13]	Kazakhstan	UMIC	2009, USD	Rota	Societal	Yes	IP
Alvis-Guzman et al., 2013 [17]	Colombia	UMIC	2010, USD	Diarrhea	Societal	No	Both but does not distinguish OP/IP
Alkoshi et al., 2015 [47]	Libya	UMIC	2013, USD	Rota	Societal	No	Both but does not distinguish OP/IP

### Cost of diarrhea illness in children

Table 2 summarizes cost estimates derived from the empirical literature in 2015 USD. The number of data points from the literature used to inform the estimates is represented by N. The mean (unweighted) cost of illness per outpatient episode of diarrhea in children across low- and middle-income countries was $36.56 (median $15.73; range $4.30 – $145.47). For inpatient care, the average cost per episode was approximately $159.90 (median $85.85; range $41.01 – $538.33). The average total direct cost and average indirect cost for outpatient care were roughly equal, whereas the median values of total direct cost was twice the median value of the indirect cost. For inpatient care, the average total direct cost was roughly 3 times the average indirect cost, but the difference was roughly four-fold for the median values. Based on studies that included but did not distinguish between outpatient or inpatient cost in reporting, the average cost of diarrhea care was about $80.50 (median $7.28; range $2.62 – $416.72).

**Table 2:** Summary of diarrhea cost of illness estimates from literature review (in 2015 USD)

	Direct medical cost	Direct non-medical cost	Total direct cost	Indirect cost	Total cost per episode
**Outpatient**					
*N*	*9*	*8*	*10*	*7*	*6*
Mean	12.54	4.77	15.52	14.02	36.56
Median	6.64	2.98	7.23	3.48	15.73
Min	0.92	0.47	1.42	1.66	4.30
Max	58.23	18.87	77.10	68.37	145.47
**Inpatient**					
*N*	*15*	*13*	*16*	*12*	*11*
Mean	117.58	16.99	116.79	39.19	159.90
Median	64.27	14.21	73.66	19.23	85.85
Min	24.24	1.18	29.99	2.47	41.01
Max	341.27	39.75	359.34	178.99	538.33
**Unspecified In/out-patient**					
*N*	*10*	*9*	*10*	*9*	*9*
Mean	39.55	11.33	56.97	17.14	80.05
Median	2.84	0.72	3.37	3.58	7.28
Min	1.22	0.24	1.70	0.91	2.62
Max	299.58	91.13	390.72	74.94	416.72

The observed variation in cost estimates was large across different health care settings in which care was sought. Data points stratified by facility types were too sparse to generate any conclusive evidence. Nevertheless, for outpatient, the total direct cost of illness reported averaged at $3.70, $33.79, and $31.86 in primary, secondary, and tertiary level health care settings, respectively. For inpatient care, the total direct costs averaged at $30.70, $144.54, and $154.89 in primary, secondary, and tertiary level hospital, respectively (see Additional file 2).

### Comparison of cost estimates

We assessed the intra-country consistency of estimates for countries with multiple costing studies using direct cost estimates, as they were the only indicator reported consistently across studies. We were only able to make within-country comparisons in a few countries. Multiple studies from Bangladesh, Bolivia, and India reported direct medical costs for inpatient care.

For Bangladesh, the reported estimates of total direct cost across *(not distinguished by Inpatient or Outpatient)* three studies were $1.70 [29], $3.49 [43], and $22.69 [36]. The higher estimates were for a tertiary-level hospital, whereas the lower estimates were from a rural setting covering costs of primary care.

In Bolivia, the two publications from the same group of authors [18, 44], which use the same data source but with separate sub-group analyses reveal variations in direct costs across private versus public facilities and across urban and rural areas. Both direct medical and non-medical costs for outpatient care in rural settings were much higher than for inpatient care. Based on their analysis, higher variation was observed in direct medical outpatient care compared to inpatient care; direct medical inpatient costs were relatively consistent across settings, with the higher estimate being only 24% higher than the lower estimate. However, the higher direct medical outpatient cost estimate was more than 2.7 times the lower outpatient cost estimate.

In India, different facility types appear to play a role in cost differences. The total direct cost for outpatient care in India ranged from $2.86 [39] in a primary care setting to $21.27 [41] in a tertiary care setting. For inpatient care, the total direct cost estimates range from $17.09 [39] in a primary hospital to $197.92 [41] in a tertiary hospital.

### Modelled estimates of cost of illness for diarrhea

To inform modelling, we used the cost structure as observed from the literature along with other information as outlined in Fig. 2. Of the 25 studies examined in the diarrhea cost of illness literature review, 18 studies [13, 16, 18, 29–34, 36, 38, 39, 41–45, 47] reported both direct medical and direct non-medical costs for either inpatient or outpatient care. Using this data, we calculated direct medical cost share as a proportion of total direct costs. Across all studies, on average, direct medical costs accounted for 79% of the total direct cost of treatment. Segregating by type of care, the average direct medical cost share was about 83% (median 85%) for inpatient and 74% (median 75%) for outpatient care. Due to sparseness of data in the literature by country income group or geographic categories, we applied an average cost share for inpatient and outpatient care observed in the literature across all countries to inform model estimates. A summary of the modelled estimates of costs of diarrhea illness by country income group is presented in Table 3.

**Table 3:** Estimated cost of diarrhea illness in children by income group (in 2015 USD)

	Inpatient				Outpatient			
	Total	Direct medical	Direct non-medical	Indirect	Total	Direct medical	Direct non-medical	Indirect
All countries (*N* = 137)								
Mean	260.60	144.83	29.84	85.94	56.41	9.23	3.19	43.99
Weighted mean	216.36	112.41	23.16	80.79	52.16	8.03	2.77	41.36
Maximum	1225.36	740.48	152.57	332.31	201.91	25.61	8.83	170.11
Minimum	23.77	8.18	1.69	6.38	8.81	3.49	1.20	3.26
Median	177.20	88.87	18.31	69.39	47.56	7.83	2.70	35.52
SD	243.20	146.77	30.24	71.56	42.64	4.96	1.71	36.63
Low income countries (*N* = 34)								
Mean	45.75	25.67	5.29	14.79	14.02	4.80	1.65	7.57
Weighted mean	44.23	24.40	5.03	14.80	13.94	4.73	1.63	7.58
Maximum	157.71	103.93	21.41	32.36	29.09	9.31	3.21	16.57
Minimum	36.60	8.18	1.69	24.56	18.38	3.49	1.20	12.57
Median	44.02	24.23	4.99	14.52	13.36	4.74	1.63	7.43
SD	22.15	15.06	3.10	4.95	3.51	0.88	0.30	2.53
Lower-middle income countries (*N* = 47)								
Mean	143.09	74.15	15.28	53.66	36.88	7.00	2.41	27.47
Weighted mean	116.59	57.33	11.81	47.45	32.58	6.17	2.13	24.29
Maximum	312.31	205.22	42.28	97.10	63.86	13.43	4.63	49.71
Minimum	36.60	8.18	1.69	24.56	18.38	3.49	1.20	12.57
Median	134.77	68.55	14.12	48.67	35.94	6.76	2.33	24.92
SD	66.70	42.19	8.69	21.81	12.75	1.91	0.66	11.16
Upper-middle income countries (*N* = 56)								
Mean	489.67	276.49	56.97	156.22	98.54	13.81	4.76	79.97
Weighted mean	491.10	259.70	53.51	177.89	108.84	13.22	4.56	91.06
Maximum	1225.36	740.48	152.57	332.31	201.91	25.61	8.83	170.11
Minimum	189.45	62.75	12.93	75.83	51.95	5.66	1.95	38.82
Median	452.14	250.05	51.52	144.59	93.59	13.06	4.50	74.01
SD	220.39	144.63	29.80	56.82	33.45	4.42	1.53	29.08

*N* represents the number of countries in each income category. The weighted mean is the mean value weighted by the country population under 5 years old (for year 2018) in each income group

**Fig. 3:**
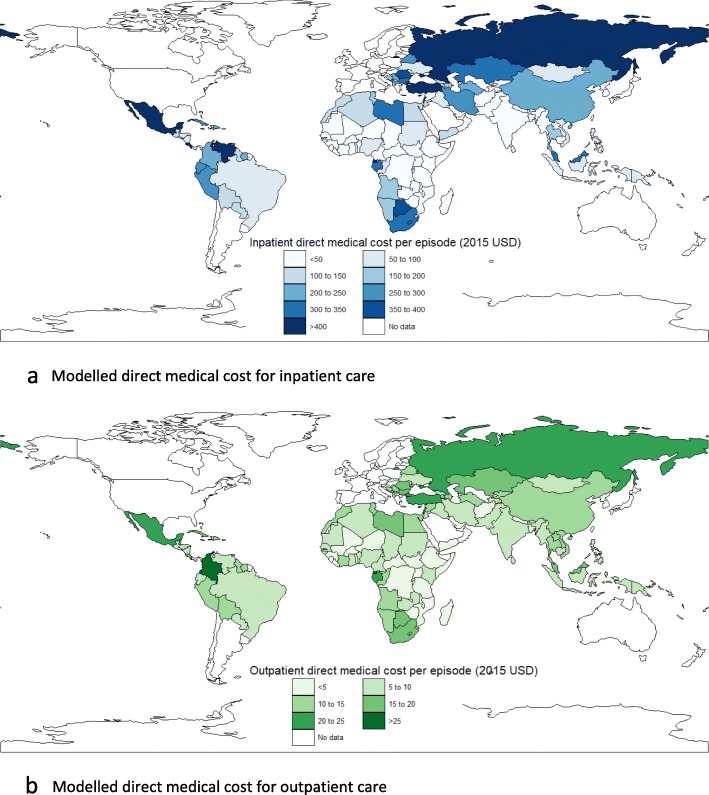
Modelled estimates of direct medical cost for inpatient and outpatient care

Based on our modelled estimates, the weighted average total cost per episode of inpatient diarrhea across all countries was $216.36. For inpatient cases, the average cost per episode for LICs, LMICs, and UMIC was estimated at $44.23, $116.59, and $491.10, respectively. Similarly, the weighted average cost per outpatient episode was estimated to be approximately $52.16. For outpatient cases, the weighted average cost for LICs, LMICs, and UMICs was $13.94, $32.58, and $108.84, respectively. The weighted average direct medical cost per inpatient visit ranged was $112.41 (range $24.40 to $259.70), while the weighted average direct medical cost per outpatient visit was $8.03 (range $4.73 to $13.22). The highest total cost per hospitalization was for UMICs at $1225.36. Cross country variations in modelled estimates of direct medical cost are shown in Fig. 3. Country-specific modelled estimates of cost of illness are provided in Additional file 3.

As a sensitivity check to the cost estimates generated using the WHO-CHOICE service delivery unit costs, we additionally evaluated the cost of diarrhea for both inpatient and outpatient care using the unit cost estimates from the IHME [27]. In general, the unit cost estimates from the IHME were higher than that for the unit costs estimate from the WHO-CHOICE. The correlation coefficient between the cross-country unit cost estimates from the two sources were positive and moderate (Pearson’s correlation 0.41 for inpatient unit cost and 0.63 for outpatient unit costs). Across all countries, on average, the cost per bed day from IHME are roughly 6 times higher and the cost per visit were about 3 times higher compared to the WHO-CHOICE unit costs.

In comparing the two sets of modelled average estimates with the average estimate from the literature, we find that the average cost per episode estimates were more closely aligned with the literature when using the WHO-CHOICE unit costs. The average difference in average cost per episode between the literature estimate and that derived using WHO-CHOICE unit cost was about 35% (population weighted) to 63% (unweighted) for inpatients; whereas the same using the unit costs from the IHME were in the order of 357% (population weighted) to 223% (unweighted). The differences in case of outpatient cost per episode is much less pronounced (see Table 4).

**Table 4:** Comparison of average estimates from literature and that modeled using unit cost inputs from different sources

Source	Inputs	Average cost per episode		% difference in cost per episode	
		IP	OP	IP	OP
Literature	Not applicable	159.90	36.56		
Modelled using WHO-CHOICE unit cost	Population unweighted average	260.60	56.41	−63%	−54%
	Population weighted average	216.36	52.16	−35%	−43%
Modelled using IHME unit cost	Population unweighted average	730.33	64.73	−357%	−77%
	Population weighted average	516.76	58.63	−223%	−60%

### Comparison of modelled and literature estimates

**Fig. 4:**
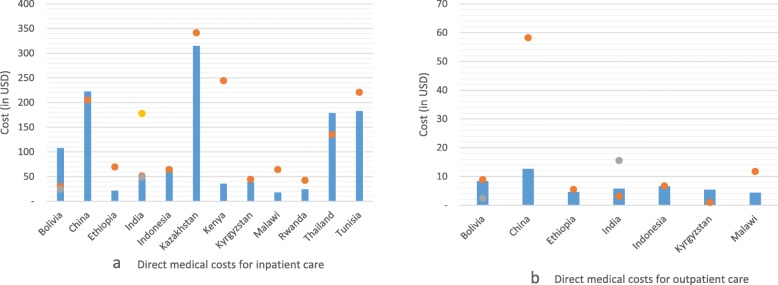
Comparison of modelled and empirical estimates of direct medical costs across countries

We compared the estimates to check consistency across modelled and empirical estimates. Figure 4 presents a comparison between direct medical costs derived from the two sources. In each figure, the bars represent modelled estimates using the WHO-CHOICE service delivery inputs and the circles represent empirical estimates. The figures show that, apart from a few observations such as the cost of inpatient care in Bolivia or Kenya (a) and the cost of outpatient care in China (b) the other estimates show general consistency between modelled and empirical estimates. In the 12 countries with primary data, there was reasonable agreement between our modelled estimates of direct inpatient care and the reported data (Pearson correlation coefficient > .75). In general, the modeled estimates are similar to the literature estimates in more than half of the comparisons.

Similar figures that compare cost estimates from 3 sources: a. empirical estimates from the literature; b. modelled estimates using the WHO-CHOICE service delivery unit costs as inputs; and c. modelled estimates using the IHME service delivery unit costs as inputs, are included in the Additional file 6.

## Discussion

We undertook a systematic review of cost of diarrhea illness among children in low-and middle-income countries. Twenty-five studies from 20 countries were included in the analysis. We found that the average costs of outpatient and inpatient diarrhea care per episode were approximately $40 and $160, respectively; the range of estimates, however, varies widely between studies. Several studies included aggregated costs of outpatient and inpatient care and did not report costs separately; estimates based on these studies alone suggest the average cost of illness per episode, to be about $80.

We found considerable variation in cost estimates reported in the literature. This variation can be attributed to the methodological differences across studies, particularly to variation in the perspective undertaken by studies and cost categorization. While most of the studies included in the systematic review reported costs from societal perspective, reflecting burden to households, a few studies reported costs from hospital or payer perspective. Further, there was considerable heterogeneity in estimates in the literature based on settings from which costing data were gathered and the health facilities in which care for diarrhea was sought. We also find a lack of standardized reporting of the cost items, a feature common across costing studies [50], adding to the issue of measurement bias. The cost of treatment could vary within and between countries based on the capabilities, and resources available for treatment in those settings leading to some heterogeneity in reported costs [50]. Nevertheless, the range in estimates are consistent with findings reported in some earlier reviews [20].

Using the ratio of direct medical to total direct costs for inpatient and outpatient care from the literature, we modelled the cost of diarrhea care across 137 low- and middle-income countries. Our model estimates an weighted average cost of approximately $216 (median $177) and $52 (median $47) per episode of diarrhea among children in inpatient and outpatient settings, respectively, across all 137 countries with a considerable range in average cost estimates across country income groups.

On average, across all countries, our modelled weighted average outpatient cost per episode was 43% higher than the average estimate from the literature. The weighted average inpatient cost per episode from the model was roughly 35% higher than that derived from the literature. Although there are notable discrepancies between the modelled and empirical estimates, there was a reasonable agreement between modelled estimates and the reported data (Pearson’s correlation coefficient > .75). However, it should be noted that the two sets of estimates are not directly comparable for various reasons. The empirical estimates are derived from only a handful of countries representing limited geography and heterogeneous health care settings, as well as varying severity of illness. The modelled estimates, on the other hand, assume a representative primary healthcare setting for outpatient care and a secondary hospital for inpatient care.

We found considerable differences in cost estimates when using the service delivery unit costs as inputs from two different sources, WHO-CHOICE and IHME (see Additional file 6). The average cost estimates per episode are much higher when using the IHME unit cost estimates. There may be numerous reasons for these differences in unit cost estimates including the time when these data were estimated, as well as the inputs that went into generating these estimates. However, discussing the difference between the two sources is beyond the scope of this paper.

Recognizing the sparseness in empirical data, the modelled estimates in this study were generated to help fill this data gap. Overall, we find conformity of our modelled cost estimates using the WHO-CHOICE inputs with the evidence generated from the systematic review. Our modelled estimates are not meant to precisely represent costs in any single country, however, in the event of lack of empirical cost of illness data for specific countries, our modelled estimates are useful to inform countries of the value of diarrhea prevention and for planning purposes.

As with any study, a number of assumptions and limitations should be considered in interpreting these findings. While our literature search was designed to comprehensively identify recent empirical evidence on diarrheal disease costs, some articles may have been inadvertently overlooked. In particular, we excluded studies from the high-income countries as well as those with data collection before year 2006. We recognize that some of those excluded studies may have had representative, high quality data to contribute to the literature review. Further, we focused our search on general diarrhea rather than a specific pathogen, but included rotavirus due to the number of known studies targeting this pathogen. We recognize this as a potential weakness, but there is some evidence supporting the hypothesis that costs may not differ greatly by pathogen once severity has been considered. However, this has not been demonstrated for direct medical costs [51].

We did not formally assess risk of bias of individual studies and this is a limitation to our analysis. Nonetheless, we followed strict guideline for the review, and also methodologically characterized each study included in the analysis to obtain harmonized and comparable measures of primary outcomes. This limits our full understanding of the direction of bias.

In addition, we have estimated indirect costs using GDP per capita as a proxy for the value of caretaker time. While this is a uniform measure that can be applied across countries, it has the potential to overstate indirect costs, particularly in low-income countries where much of the labor force is in the informal sector and the GDP per capita may not accurately reflect the value of productivity costs. Finally, while we view the estimates we have generated as useful inputs for planning, scaling up, and/or evaluating interventions targeting diarrhea, we also believe that country-specific modelled estimates should be used cautiously. We would be hesitant to default to modelled estimates when high-quality empirical estimates are available.

## Conclusions

Our study sought first to undertake a systematic review of literature on diarrhea cost of illness in children under 5 years and, second, to generate modelled estimates of diarrhea cost of illness for children in 137 low-and middle-income countries. Our modelled estimates generally correspond to estimates observed in the literature, with a few exceptions. In the absence of country-specific estimates, those generated in our model could serve as useful inputs for planning and prioritizing appropriate health interventions for childhood diarrheal diseases in LMICs.

## Supplementary information

**Additional file 1.** Search terms used in systematic review.

**Additional file 2.** Diarrhea cost of illness (2015 USD) from literature by level of facility.

**Additional file 3.** Modelled cost of illness (2015 USD) estimates by country, using WHO-CHOICE service delivery unit cost estimates.

**Additional file 4.** Modelled cost of illness (2015 USD) estimates by country, using IHME service delivery unit cost estimates.

**Additional file 5.** Modelled cost of illness (2015 USD) estimates by country, excluding cost associated with oral rehydrated solution (ORS).

**Additional file 6.** Comparison of modelled and empirical estimates of direct medical costs across countries.

## Data Availability

All data generated or analysed during this study are included in this published article [and its supplementary information files].

## Abbreviations

USD: US dollar
GDPpc: Gross Domestic Product per capita
