# Supplementary material for: Cost of illness for childhood diarrhea in low- and middle-income countries: a systematic review of evidence and modelled estimates
Source: BMC Public Health. 2020 May 5;20:619. doi: 10.1186/s12889-020-08595-8 (PMC7201538; doi:10.1186/s12889-020-08595-8)
Supplement: Supplementary file 1 — Additional file 1. Search terms used in systematic review. [file 12889_2020_8595_MOESM1_ESM.docx]

**Supplemental materials**

**S1 Appendix: Search terms used in systematic review**

|  |  |  | **Search terms** |
| --- | --- | --- | --- |
| **Domain** | **Sub-category** | **Search** | **Pubmed** |
| Economic burden | Direct  Indirect healthcare cost | 1 | Cost OR economic OR “cost of illness” OR “burden of illness” OR “illness burden” OR “economic burden” OR expenditure OR “quality of life” OR funding |
| Disease | Diarrhea | 2 | diarrhea* OR diarrhea OR diarrhoea* OR diarrhoea |
|  |  | 3 | Rotavirus OR “Rotaviruses” OR “Rotavirus Infections” OR “Infection, Rotavirus” OR “Infections, Rotavirus” OR “Rotavirus Infection” |
| LMIC | - Low-income or middle-income - Less developed - under developed - Developing Countries | 4 | ((Low-income or middle-income or developing or less developed or under developed or poor) AND (countr* or nation*)) OR (Afghanistan OR Albania OR Algeria OR Angola OR American Samoa OR Armenia OR Bangladesh OR Belarus OR Belize OR (Bosnia and Herzegovina) OR Botswana OR Brazil OR Burundi OR Mexico OR Benin OR Micronesia OR Bhutan OR Libya OR Malaysia OR Maldives OR Mali OR Azerbaijan OR Marshall Islands OR Mauritania OR Macedonia OR Madagascar OR Malawi OR Mauritius OR Moldova OR Bolivia OR Mongolia OR Montenegro OR Morocco OR Mozambique OR Bulgaria OR Myanmar OR Burkina Faso OR Namibia OR Nepal OR Cabo Verde OR Nicaragua OR Cambodia OR Niger OR Cameroon OR Nigeria OR Central African Republic OR Pakistan OR Chad OR Palau OR China OR Panama OR Colombia OR Papua New Guinea OR Comoros OR Paraguay OR Congo OR Peru OR Congo OR Philippines OR Costa Rica OR Romania OR Cote d'Ivoire OR Ivory Coast OR Rwanda OR Cuba OR Samoa OR Djibouti OR (Sao Tome and Principe) OR Dominica OR Senegal OR Dominican Republic OR Serbia OR Ecuador OR Seychelles OR Egypt OR Sierra Leone OR El Salvador OR Solomon Islands OR Eritrea OR Somalia OR Ethiopia OR South Africa OR Fiji OR South Sudan OR Gabon OR Sri Lanka OR Gambia OR Saint Lucia OR Georgia OR (Saint Vincent and the Grenadines) OR Ghana OR Sudan OR Grenada OR Suriname OR Guatemala OR Swaziland OR Guinea OR Syrian Arab Republic OR Guinea-Bissau OR Tajikistan OR Guyana OR Tanzania OR Haiti OR Thailand OR Honduras OR Timor-Leste OR Hungary OR Togo OR India OR Tonga OR Indonesia OR Tunisia OR Iran OR Turkey OR Iraq OR Turkmenistan OR Jamaica OR Tuvalu OR Jordan OR Uganda OR Kazakhstan OR Ukraine OR Kenya OR Uzbekistan OR Kiribati OR Vanuatu OR Korea OR Venezuela OR Kosovo OR Vietnam OR Kyrgyz Republic OR (West Bank and Gaza) OR Lao PDR OR Yemen OR Lebanon OR Zambia OR Lesotho OR Zimbabwe OR Liberia OR (Antigua and Barbuda) OR Argentina OR Bahrain OR Barbados OR Chile OR Croatia OR (Czech Republic) OR (Equatorial Guinea) OR Estonia OR Hungary OR (Isle of Man) OR Latvia OR Lithuania OR Oman OR Poland OR (Puerto Rico) OR (Russian Federation) OR Russia OR (Saudi Arabia) OR Seychelles OR (Slovak Republic) OR (St Kitts and Nevis) OR (Trinidad and Tobago) OR Uruguay OR Venezuela) OR *Developing Countries/* |
| Combined search in titles, abstracts and keywords | |  | #1 AND (#2 OR #3 OR #4) |
