# Supplementary material for: Cost of illness for childhood diarrhea in low- and middle-income countries: a systematic review of evidence and modelled estimates
Source: BMC Public Health. 2020 May 5;20:619. doi: 10.1186/s12889-020-08595-8 (PMC7201538; doi:10.1186/s12889-020-08595-8)
Supplement: Supplementary file 2 — Additional file 2. Diarrhea cost of illness (2015 USD) from literature by level of facility. [file 12889_2020_8595_MOESM2_ESM.docx]

**S2 Appendix: Diarrhea cost of illness (2015 USD) from literature by level of facility**

|  |  | **Primary level facility** | | | | | **Secondary level facility** | | | | | **Tertiary level facility** | | | | |
| --- | --- | --- | --- | --- | --- | --- | --- | --- | --- | --- | --- | --- | --- | --- | --- | --- |
|  |  | **Direct medical** | **Direct non-medical** | **Total Direct** | **Indirect cost** | **Total cost per episode** | **Direct medical** | **Direct non-medical** | **Total Direct** | **Indirect cost** | **Total cost per episode** | **Direct medical** | **Direct non-medical** | **Total Direct** | **Indirect cost** | **Total cost per episode** |
| **Outpatient** | N | 2 | 2 | 4 | 2 | 2 | 4 | 4 | 4 | 4 | 4 | 5 | 4 | 3 | 2 | 1 |
|  | Mean | 3.90 | 0.49 | 3.70 | 2.13 | 5.80 | 22.59 | 8.83 | 33.79 | 21.60 | 53.96 | 19.00 | 6.83 | 31.86 | 38.37 | 144.86 |
|  | Median | 3.90 | 0.49 | 3.72 | 2.13 | 5.80 | 13.61 | 5.09 | 18.08 | 9.95 | 27.12 | 3.95 | 3.07 | 21.27 | 38.37 | 144.86 |
|  | Min | 2.24 | 0.35 | 1.42 | 1.38 | 4.30 | 2.41 | 1.85 | 14.97 | 4.66 | 15.74 | 0.92 | 0.38 | 4.33 | 1.86 | 144.86 |
|  | Max | 5.56 | 0.62 | 5.91 | 2.87 | 7.30 | 60.74 | 23.29 | 84.03 | 61.84 | 145.87 | 55.62 | 14.36 | 69.97 | 74.89 | 144.86 |
| **Inpatient** | N | 2 | 2 | 4 | 2 | 1 | 6 | 5 | 5 | 6 | 5 | 9 | 8 | 9 | 6 | 6 |
|  | Mean | 31.90 | 3.74 | 30.70 | 14.41 | 62.00 | 111.70 | 19.05 | 144.54 | 45.00 | 173.66 | 143.92 | 18.28 | 154.89 | 55.05 | 211.79 |
|  | Median | 31.90 | 3.74 | 25.76 | 14.41 | 62.00 | 74.23 | 14.11 | 120.68 | 22.70 | 97.04 | 81.27 | 18.95 | 106.92 | 19.93 | 151.33 |
|  | Min | 15.92 | 1.17 | 17.09 | 7.82 | 62.00 | 24.25 | 5.76 | 39.93 | 10.70 | 41.01 | 44.46 | 1.18 | 58.66 | 2.47 | 65.63 |
|  | Max | 47.87 | 6.32 | 54.19 | 21.00 | 62.00 | 332.19 | 50.90 | 383.09 | 155.31 | 538.40 | 341.27 | 38.93 | 359.34 | 178.99 | 538.33 |
| **Unspecified in/out-patient** | N | 1 | 0 | 0 | 0 | 0 | 3 | 2 | 2 | 2 | 2 | 2 | 1 | 1 | 1 | 1 |
|  | Mean | 20.41 | - | - | - | - | 147.46 | 56.31 | 263.36 | 50.47 | 313.83 | 79.26 | 6.96 | 22.69 | 33.45 | 56.15 |
|  | Median | 20.41 | - | - | - | - | 114.51 | 56.31 | 263.36 | 50.47 | 313.83 | 79.26 | 6.96 | 22.69 | 33.45 | 56.15 |
|  | Min | 20.41 | - | - | - | - | 28.30 | 21.49 | 136.00 | 26.00 | 210.93 | 15.73 | 6.96 | 22.69 | 33.45 | 56.15 |
|  | Max | 20.41 | - | - | - | - | 299.58 | 91.13 | 390.72 | 74.94 | 416.72 | 142.79 | 6.96 | 22.69 | 33.45 | 56.15 |
