# Supplementary material for: Cost of illness for childhood diarrhea in low- and middle-income countries: a systematic review of evidence and modelled estimates
Source: BMC Public Health. 2020 May 5;20:619. doi: 10.1186/s12889-020-08595-8 (PMC7201538; doi:10.1186/s12889-020-08595-8)
Supplement: Supplementary file 3 — Additional file 3. Modelled cost of illness (2015 USD) estimates by country, using WHO-CHOICE service delivery unit cost estimates. [file 12889_2020_8595_MOESM3_ESM.docx]

**S3 Appendix: Modelled cost of illness (2015 USD) estimates by country, using WHO CHOICE service delivery unit cost estimates**

| **Country** | **Inpatient** | **Outpatient** | **Inpatient-Direct Medical** | **Inpatient-Direct Non-Medical** | **Inpatient-Indirect** | **Outpatient-Direct Medical** | **Outpatient-Direct Non-Medical** | **Outpatient-Indirect** |
| --- | --- | --- | --- | --- | --- | --- | --- | --- |
| Afghanistan | 30.82 | 12.49 | 14.22 | 2.93 | 13.68 | 4.08 | 1.41 | 7.00 |
| Albania | 298.60 | 60.11 | 172.31 | 35.50 | 90.79 | 10.13 | 3.50 | 46.48 |
| Algeria | 251.59 | 60.40 | 129.34 | 26.65 | 95.60 | 8.53 | 2.94 | 48.94 |
| American Samoa | 599.08 | 154.54 | 276.49 | 56.97 | 265.62 | 13.81 | 4.76 | 135.97 |
| Angola | 312.31 | 63.86 | 180.69 | 37.23 | 94.39 | 11.56 | 3.99 | 48.32 |
| Armenia | 230.74 | 53.09 | 124.74 | 25.70 | 80.30 | 8.91 | 3.08 | 41.10 |
| Azerbaijan | 340.03 | 78.29 | 177.04 | 36.48 | 126.52 | 10.06 | 3.47 | 64.76 |
| Bangladesh | 65.20 | 21.11 | 30.94 | 6.37 | 27.89 | 5.08 | 1.75 | 14.27 |
| Belarus | 452.09 | 84.50 | 265.05 | 54.61 | 132.43 | 12.42 | 4.29 | 67.79 |
| Belize | 387.09 | 74.77 | 227.86 | 46.95 | 112.28 | 12.86 | 4.44 | 57.48 |
| Benin | 53.07 | 15.73 | 29.46 | 6.07 | 17.54 | 5.02 | 1.73 | 8.98 |
| Bhutan | 175.52 | 41.44 | 94.85 | 19.54 | 61.12 | 7.55 | 2.60 | 31.29 |
| Bolivia | 200.59 | 47.56 | 107.61 | 22.17 | 70.81 | 8.41 | 2.90 | 36.25 |
| Bosnia and Herzegovina | 330.78 | 64.90 | 193.18 | 39.80 | 97.79 | 11.03 | 3.81 | 50.06 |
| Botswana | 609.85 | 96.99 | 384.29 | 79.18 | 146.37 | 16.40 | 5.66 | 74.93 |
| Brazil | 292.95 | 109.85 | 77.31 | 15.93 | 199.71 | 5.66 | 1.95 | 102.23 |
| Bulgaria | 543.33 | 101.52 | 317.06 | 65.33 | 160.95 | 14.23 | 4.91 | 82.39 |
| Burkina Faso | 42.55 | 13.26 | 24.02 | 4.95 | 13.57 | 4.69 | 1.62 | 6.95 |
| Burundi | 23.77 | 8.81 | 14.42 | 2.97 | 6.38 | 4.12 | 1.42 | 3.26 |
| Cabo Verde | 184.64 | 47.05 | 94.32 | 19.43 | 70.89 | 8.01 | 2.76 | 36.29 |
| Cambodia | 71.70 | 20.85 | 37.34 | 7.69 | 26.67 | 5.35 | 1.85 | 13.65 |
| Cameroon | 78.73 | 21.94 | 42.05 | 8.66 | 28.01 | 5.65 | 1.95 | 14.34 |
| Central African Republic | 33.99 | 10.15 | 22.02 | 4.54 | 7.44 | 4.72 | 1.63 | 3.81 |
| Chad | 50.63 | 15.70 | 27.18 | 5.60 | 17.85 | 4.88 | 1.68 | 9.14 |
| China | 453.93 | 112.07 | 222.40 | 45.82 | 185.70 | 12.65 | 4.36 | 95.06 |
| Colombia | 419.42 | 98.36 | 232.20 | 47.84 | 139.37 | 20.08 | 6.93 | 71.35 |
| Comoros | 50.61 | 15.25 | 28.27 | 5.82 | 16.51 | 5.06 | 1.74 | 8.45 |
| DRC | 28.11 | 11.05 | 14.60 | 3.01 | 10.50 | 4.22 | 1.46 | 5.37 |
| Congo, Rep. | 120.16 | 30.39 | 64.30 | 13.25 | 42.60 | 6.38 | 2.20 | 21.81 |
| Costa Rica | 844.04 | 161.00 | 484.97 | 99.93 | 259.14 | 21.07 | 7.27 | 132.65 |
| Côte d'Ivoire | 80.09 | 23.97 | 39.71 | 8.18 | 32.20 | 5.57 | 1.92 | 16.48 |
| Cuba | 518.84 | 114.34 | 284.22 | 58.56 | 176.06 | 18.00 | 6.21 | 90.13 |
| Djibouti | 112.88 | 31.63 | 56.48 | 11.64 | 44.76 | 6.48 | 2.24 | 22.92 |
| Dominica | 764.41 | 112.41 | 498.02 | 102.61 | 163.77 | 21.24 | 7.33 | 83.84 |
| Dominican Republic | 435.14 | 93.14 | 237.37 | 48.91 | 148.86 | 12.59 | 4.34 | 76.20 |
| Ecuador | 476.26 | 91.67 | 276.49 | 56.97 | 142.80 | 13.81 | 4.76 | 73.10 |
| Egypt, Arab Rep. | 211.43 | 53.25 | 106.33 | 21.91 | 83.19 | 7.93 | 2.74 | 42.58 |
| El Salvador | 129.96 | 55.53 | 27.25 | 5.61 | 97.10 | 4.33 | 1.49 | 49.71 |
| Equatorial Guinea | 1,225.36 | 201.91 | 740.48 | 152.57 | 332.31 | 23.64 | 8.16 | 170.11 |
| Eritrea | 28.99 | 11.87 | 13.65 | 2.81 | 12.53 | 4.05 | 1.40 | 6.41 |
| Ethiopia | 40.22 | 13.47 | 21.54 | 4.44 | 14.25 | 4.59 | 1.58 | 7.29 |
| Fiji | 279.95 | 71.80 | 137.47 | 28.32 | 114.16 | 9.93 | 3.43 | 58.44 |
| Gabon | 612.26 | 117.94 | 349.92 | 72.10 | 190.24 | 15.28 | 5.27 | 97.39 |
| Gambia | 60.87 | 13.20 | 41.48 | 8.55 | 10.85 | 5.68 | 1.96 | 5.56 |
| Georgia | 212.91 | 55.22 | 104.84 | 21.60 | 86.46 | 8.14 | 2.81 | 44.26 |
| Ghana | 75.66 | 23.49 | 36.60 | 7.54 | 31.52 | 5.47 | 1.89 | 16.14 |
| Grenada | 701.25 | 133.63 | 405.66 | 83.58 | 212.00 | 18.67 | 6.44 | 108.53 |
| Guatemala | 238.94 | 58.33 | 123.63 | 25.47 | 89.83 | 9.17 | 3.16 | 45.99 |
| Guinea | 36.12 | 12.24 | 19.81 | 4.08 | 12.23 | 4.45 | 1.53 | 6.26 |
| Guinea-Bissau | 42.77 | 13.16 | 24.53 | 5.05 | 13.19 | 4.77 | 1.64 | 6.75 |
| Guyana | 262.09 | 63.39 | 138.55 | 28.55 | 94.99 | 10.98 | 3.79 | 48.62 |
| Haiti | 57.03 | 16.84 | 31.68 | 6.53 | 18.83 | 5.35 | 1.85 | 9.64 |
| Honduras | 175.02 | 40.81 | 96.86 | 19.96 | 58.20 | 8.19 | 2.82 | 29.79 |
| India | 96.81 | 26.53 | 49.87 | 10.28 | 36.67 | 5.77 | 1.99 | 18.77 |
| Indonesia | 159.68 | 48.25 | 68.55 | 14.12 | 77.02 | 6.56 | 2.26 | 39.42 |
| Iran, Islamic Rep. | 435.95 | 80.17 | 257.61 | 53.08 | 125.26 | 11.93 | 4.12 | 64.12 |
| Iraq | 189.45 | 66.85 | 62.75 | 12.93 | 113.77 | 6.40 | 2.21 | 58.24 |
| Jamaica | 393.63 | 77.52 | 228.96 | 47.17 | 117.50 | 12.91 | 4.45 | 60.15 |
| Jordan | 241.33 | 69.00 | 105.83 | 21.81 | 113.69 | 8.03 | 2.77 | 58.20 |
| Kazakhstan | 621.92 | 143.39 | 315.12 | 64.93 | 241.87 | 14.55 | 5.02 | 123.82 |
| Kenya | 74.94 | 23.51 | 35.86 | 7.39 | 31.68 | 5.42 | 1.87 | 16.22 |
| Kiribati | 130.61 | 27.58 | 81.12 | 16.71 | 32.78 | 8.03 | 2.77 | 16.78 |
| Korea, Dem. People's Rep. | 45.75 | 14.02 | 25.67 | 5.29 | 14.79 | 4.80 | 1.65 | 7.57 |
| Kosovo | 171.18 | 51.26 | 74.15 | 15.28 | 81.75 | 7.00 | 2.41 | 41.85 |
| Kyrgyzstan | 72.51 | 20.29 | 39.07 | 8.05 | 25.39 | 5.42 | 1.87 | 13.00 |
| Lao PDR | 96.24 | 29.14 | 45.10 | 9.29 | 41.85 | 5.74 | 1.98 | 21.42 |
| Lebanon | 776.15 | 122.17 | 489.99 | 100.96 | 185.21 | 20.34 | 7.02 | 94.81 |
| Lesotho | 67.93 | 19.81 | 35.97 | 7.41 | 24.56 | 5.38 | 1.86 | 12.57 |
| Liberia | 29.75 | 11.12 | 15.97 | 3.29 | 10.49 | 4.27 | 1.47 | 5.37 |
| Libya | 499.25 | 82.77 | 308.67 | 63.60 | 126.98 | 13.21 | 4.56 | 65.00 |
| Macedonia, FYR | 378.69 | 72.59 | 221.39 | 45.62 | 111.68 | 11.46 | 3.95 | 57.17 |
| Madagascar | 29.69 | 10.42 | 16.95 | 3.49 | 9.25 | 4.23 | 1.46 | 4.73 |
| Malawi | 30.53 | 10.24 | 18.22 | 3.75 | 8.56 | 4.36 | 1.50 | 4.38 |
| Malaysia | 633.26 | 134.75 | 338.67 | 69.78 | 224.81 | 14.63 | 5.05 | 115.08 |
| Maldives | 551.30 | 119.67 | 296.90 | 61.17 | 193.22 | 15.44 | 5.33 | 98.91 |
| Mali | 47.55 | 15.03 | 25.61 | 5.28 | 16.67 | 4.83 | 1.67 | 8.53 |
| Marshall Islands | 195.78 | 51.95 | 97.72 | 20.13 | 77.92 | 8.97 | 3.09 | 39.89 |
| Mauritania | 75.02 | 23.21 | 36.04 | 7.43 | 31.55 | 5.25 | 1.81 | 16.15 |
| Mauritius | 706.83 | 132.12 | 409.52 | 84.38 | 212.93 | 17.19 | 5.93 | 109.00 |
| Mexico | 826.68 | 133.42 | 513.61 | 105.83 | 207.24 | 20.33 | 7.01 | 106.09 |
| Micronesia, Fed. Sts. | 242.08 | 50.09 | 143.19 | 29.50 | 69.39 | 10.83 | 3.74 | 35.52 |
| Moldova | 113.46 | 30.30 | 58.81 | 12.12 | 42.53 | 6.34 | 2.19 | 21.77 |
| Mongolia | 177.20 | 55.83 | 71.22 | 14.67 | 91.31 | 6.76 | 2.33 | 46.74 |
| Montenegro | 531.37 | 94.93 | 318.31 | 65.58 | 147.48 | 14.45 | 4.98 | 75.50 |
| Morocco | 212.12 | 46.05 | 120.96 | 24.92 | 66.24 | 9.03 | 3.12 | 33.91 |
| Mozambique | 37.77 | 12.42 | 21.22 | 4.37 | 12.18 | 4.60 | 1.59 | 6.23 |
| Myanmar | 36.60 | 18.38 | 8.18 | 1.69 | 26.73 | 3.49 | 1.20 | 13.68 |
| Namibia | 303.77 | 68.90 | 162.69 | 33.52 | 107.56 | 10.29 | 3.55 | 55.06 |
| Nauru | 269.29 | 104.73 | 69.62 | 14.34 | 185.33 | 7.33 | 2.53 | 94.87 |
| Nepal | 44.64 | 14.92 | 22.83 | 4.70 | 17.11 | 4.58 | 1.58 | 8.76 |
| Nicaragua | 136.30 | 33.99 | 73.19 | 15.08 | 48.03 | 6.99 | 2.41 | 24.59 |
| Niger | 27.10 | 9.88 | 15.62 | 3.22 | 8.26 | 4.20 | 1.45 | 4.23 |
| Nigeria | 124.74 | 39.87 | 52.45 | 10.81 | 61.49 | 6.24 | 2.15 | 31.48 |
| Pakistan | 81.40 | 24.16 | 40.12 | 8.27 | 33.02 | 5.39 | 1.86 | 16.90 |
| Papua New Guinea | 147.57 | 37.25 | 79.08 | 16.29 | 52.20 | 7.83 | 2.70 | 26.72 |
| Paraguay | 264.54 | 61.01 | 141.47 | 29.15 | 93.92 | 9.62 | 3.32 | 48.08 |
| Peru | 452.19 | 89.05 | 259.93 | 53.56 | 138.71 | 13.42 | 4.63 | 71.00 |
| Philippines | 162.77 | 43.94 | 79.54 | 16.39 | 66.84 | 7.23 | 2.49 | 34.21 |
| Romania | 669.00 | 127.67 | 383.34 | 78.98 | 206.68 | 16.26 | 5.61 | 105.80 |
| Russian Federation | 840.33 | 135.60 | 518.74 | 106.88 | 214.70 | 19.10 | 6.59 | 109.91 |
| Rwanda | 45.52 | 14.61 | 24.43 | 5.03 | 16.05 | 4.76 | 1.64 | 8.22 |
| Samoa | 259.48 | 60.16 | 139.99 | 28.84 | 90.64 | 10.23 | 3.53 | 46.40 |
| São Tomé and Principe | 92.40 | 27.66 | 44.76 | 9.22 | 38.41 | 5.95 | 2.05 | 19.66 |
| Senegal | 64.15 | 17.81 | 36.02 | 7.42 | 20.70 | 5.36 | 1.85 | 10.60 |
| Serbia | 412.99 | 78.21 | 242.50 | 49.96 | 120.53 | 12.27 | 4.23 | 61.70 |
| Sierra Leone | 44.82 | 14.15 | 24.70 | 5.09 | 15.03 | 4.80 | 1.66 | 7.69 |
| Solomon Islands | 183.40 | 35.96 | 115.15 | 23.73 | 44.53 | 9.79 | 3.38 | 22.79 |
| Somalia | 43.60 | 12.92 | 25.67 | 5.29 | 12.64 | 4.80 | 1.65 | 6.47 |
| South Africa | 538.54 | 89.05 | 337.42 | 69.52 | 131.60 | 16.12 | 5.56 | 67.37 |
| South Sudan | 47.77 | 15.06 | 25.67 | 5.29 | 16.81 | 4.80 | 1.65 | 8.61 |
| Sri Lanka | 193.07 | 55.94 | 85.17 | 17.55 | 90.36 | 7.20 | 2.48 | 46.25 |
| St. Lucia | 728.71 | 118.42 | 456.60 | 94.08 | 178.03 | 20.28 | 7.00 | 91.14 |
| St. Vincent and the Grenadines | 598.09 | 102.53 | 367.32 | 75.68 | 155.09 | 17.20 | 5.93 | 79.39 |
| Sudan | 134.77 | 37.83 | 65.67 | 13.53 | 55.57 | 6.98 | 2.41 | 28.45 |
| Suriname | 508.26 | 129.50 | 240.43 | 49.54 | 218.29 | 13.20 | 4.55 | 111.74 |
| Swaziland | 195.77 | 48.37 | 101.26 | 20.86 | 73.65 | 7.94 | 2.74 | 37.70 |
| Syrian Arab Republic | 32.79 | 12.68 | 14.92 | 3.07 | 14.79 | 3.80 | 1.31 | 7.57 |
| Tajikistan | 61.88 | 17.86 | 33.64 | 6.93 | 21.31 | 5.17 | 1.78 | 10.91 |
| Tanzania | 51.85 | 16.84 | 26.22 | 5.40 | 20.23 | 4.82 | 1.66 | 10.36 |
| Thailand | 349.49 | 82.43 | 178.83 | 36.85 | 133.82 | 10.36 | 3.57 | 68.50 |
| Timor-Leste | 60.15 | 20.80 | 26.66 | 5.49 | 28.00 | 4.81 | 1.66 | 14.34 |
| Togo | 36.47 | 12.56 | 19.56 | 4.03 | 12.88 | 4.44 | 1.53 | 6.59 |
| Tonga | 283.24 | 62.95 | 156.64 | 32.27 | 94.32 | 10.90 | 3.76 | 48.28 |
| Tunisia | 308.64 | 58.98 | 182.97 | 37.70 | 87.97 | 10.37 | 3.58 | 45.03 |
| Turkey | 744.68 | 131.68 | 443.32 | 91.34 | 210.02 | 17.97 | 6.20 | 107.51 |
| Turkmenistan | 395.99 | 94.03 | 201.02 | 41.42 | 153.56 | 11.47 | 3.96 | 78.61 |
| Tuvalu | 228.31 | 52.95 | 126.43 | 26.05 | 75.83 | 10.51 | 3.62 | 38.82 |
| Uganda | 44.43 | 14.56 | 23.38 | 4.82 | 16.23 | 4.65 | 1.60 | 8.31 |
| Ukraine | 167.85 | 34.99 | 98.82 | 20.36 | 48.67 | 7.49 | 2.58 | 24.92 |
| Uzbekistan | 156.25 | 35.94 | 88.87 | 18.31 | 49.07 | 8.05 | 2.78 | 25.12 |
| Vanuatu | 312.06 | 51.11 | 205.22 | 42.28 | 64.56 | 13.43 | 4.63 | 33.05 |
| Venezuela, RB | 1,025.24 | 178.94 | 616.05 | 126.93 | 282.26 | 25.61 | 8.83 | 144.49 |
| Vietnam | 116.09 | 33.20 | 55.98 | 11.53 | 48.58 | 6.19 | 2.14 | 24.87 |
| West Bank and Gaza | 155.40 | 43.18 | 74.15 | 15.28 | 65.98 | 7.00 | 2.41 | 33.77 |
| Yemen, Rep. | 157.71 | 29.09 | 103.93 | 21.41 | 32.36 | 9.31 | 3.21 | 16.57 |
| Zambia | 63.52 | 21.95 | 27.77 | 5.72 | 30.03 | 4.89 | 1.69 | 15.37 |
| Zimbabwe | 52.23 | 17.34 | 25.67 | 5.29 | 21.27 | 4.80 | 1.65 | 10.89 |
