# Supplementary material for: Cost of illness for childhood diarrhea in low- and middle-income countries: a systematic review of evidence and modelled estimates
Source: BMC Public Health. 2020 May 5;20:619. doi: 10.1186/s12889-020-08595-8 (PMC7201538; doi:10.1186/s12889-020-08595-8)
Supplement: Supplementary file 4 — Additional file 4. Modelled cost of illness (2015 USD) estimates by country, using IHME service delivery unit cost estimates. [file 12889_2020_8595_MOESM4_ESM.docx]

**S4 Appendix: Modelled cost of illness (2015 USD) estimates by country, using IHME service delivery unit cost estimates**

| **Country** | **Inpatient** | **Outpatient** |  | **Inpatient-Direct Medical** | **Inpatient-Direct Non-Medical** | **Inpatient-Indirect** | **Outpatient-Direct Medical** | **Outpatient-Direct Non-Medical** | **Outpatient-Indirect** |
| --- | --- | --- | --- | --- | --- | --- | --- | --- | --- |
| Afghanistan | 134.61 | 9.55 |  | 100.27 | 20.66 | 13.68 | 1.89 | 0.65 | 7.00 |
| Albania | 655.67 | 62.44 |  | 468.37 | 96.51 | 90.79 | 11.87 | 4.10 | 46.48 |
| Algeria | 800.07 | 75.02 |  | 584.12 | 120.35 | 95.60 | 19.39 | 6.69 | 48.94 |
| American Samoa | 1,393.20 | 174.46 |  | 934.94 | 192.64 | 265.62 | 28.61 | 9.87 | 135.97 |
| Angola | 354.29 | 61.06 |  | 215.50 | 44.40 | 94.39 | 9.48 | 3.27 | 48.32 |
| Armenia | 543.24 | 67.14 |  | 383.85 | 79.09 | 80.30 | 19.36 | 6.68 | 41.10 |
| Azerbaijan | 1,116.30 | 89.36 |  | 820.68 | 169.10 | 126.52 | 18.29 | 6.31 | 64.76 |
| Bangladesh | 104.96 | 16.55 |  | 63.91 | 13.17 | 27.89 | 1.69 | 0.58 | 14.27 |
| Belarus | 418.05 | 77.69 |  | 236.82 | 48.80 | 132.43 | 7.35 | 2.54 | 67.79 |
| Belize | 1,001.43 | 98.37 |  | 737.25 | 151.90 | 112.28 | 30.40 | 10.49 | 57.48 |
| Benin | 114.47 | 12.15 |  | 80.37 | 16.56 | 17.54 | 2.36 | 0.81 | 8.98 |
| Bhutan | 235.51 | 37.10 |  | 144.59 | 29.79 | 61.12 | 4.32 | 1.49 | 31.29 |
| Bolivia | 526.17 | 50.37 |  | 377.56 | 77.79 | 70.81 | 10.50 | 3.62 | 36.25 |
| Bosnia and Herzegovina | 742.60 | 62.53 |  | 534.65 | 110.16 | 97.79 | 9.27 | 3.20 | 50.06 |
| Botswana | 1,152.13 | 132.89 |  | 833.93 | 171.83 | 146.37 | 43.09 | 14.87 | 74.93 |
| Brazil | 1,679.56 | 165.61 |  | 1,227.03 | 252.82 | 199.71 | 47.12 | 16.26 | 102.23 |
| Bulgaria | 553.58 | 115.85 |  | 325.56 | 67.08 | 160.95 | 24.88 | 8.58 | 82.39 |
| Burkina Faso | 102.52 | 13.13 |  | 73.75 | 15.20 | 13.57 | 4.60 | 1.59 | 6.95 |
| Burundi | 72.18 | 4.54 |  | 54.56 | 11.24 | 6.38 | 0.95 | 0.33 | 3.26 |
| Cabo Verde | 493.35 | 51.25 |  | 350.29 | 72.18 | 70.89 | 11.13 | 3.84 | 36.29 |
| Cambodia | 667.04 | 21.74 |  | 530.97 | 109.40 | 26.67 | 6.02 | 2.08 | 13.65 |
| Cameroon | 181.43 | 20.04 |  | 127.21 | 26.21 | 28.01 | 4.24 | 1.46 | 14.34 |
| Central African Republic | 36.33 | 5.17 |  | 23.95 | 4.94 | 7.44 | 1.01 | 0.35 | 3.81 |
| Chad | 139.61 | 13.06 |  | 100.96 | 20.80 | 17.85 | 2.92 | 1.01 | 9.14 |
| China | 771.21 | 138.05 |  | 485.47 | 100.03 | 185.70 | 31.96 | 11.03 | 95.06 |
| Colombia | 1,842.45 | 95.07 |  | 1,412.12 | 290.96 | 139.37 | 17.64 | 6.09 | 71.35 |
| Comoros | 99.29 | 13.24 |  | 68.64 | 14.14 | 16.51 | 3.56 | 1.23 | 8.45 |
| DRC | 58.57 | 7.64 |  | 39.86 | 8.21 | 10.50 | 1.69 | 0.58 | 5.37 |
| Congo, Rep. | 147.16 | 26.79 |  | 86.69 | 17.86 | 42.60 | 3.70 | 1.28 | 21.81 |
| Costa Rica | 3,248.23 | 260.71 |  | 2,478.43 | 510.67 | 259.14 | 95.21 | 32.85 | 132.65 |
| Côte d'Ivoire | 221.86 | 24.01 |  | 157.26 | 32.40 | 32.20 | 5.60 | 1.93 | 16.48 |
| Cuba | 1,303.64 | 128.61 |  | 934.94 | 192.64 | 176.06 | 28.61 | 9.87 | 90.13 |
| Djibouti | 493.38 | 34.70 |  | 371.97 | 76.64 | 44.76 | 8.76 | 3.02 | 22.92 |
| Dominica | 1,073.89 | 114.24 |  | 754.63 | 155.49 | 163.77 | 22.61 | 7.80 | 83.84 |
| Dominican Republic | 974.68 | 131.31 |  | 684.73 | 141.09 | 148.86 | 40.97 | 14.13 | 76.20 |
| Ecuador | 1,299.80 | 111.19 |  | 959.34 | 197.67 | 142.80 | 28.32 | 9.77 | 73.10 |
| Egypt, Arab Rep. | 306.85 | 49.77 |  | 185.45 | 38.21 | 83.19 | 5.34 | 1.84 | 42.58 |
| El Salvador | 2,350.29 | 107.06 |  | 1,868.25 | 384.94 | 97.10 | 42.64 | 14.71 | 49.71 |
| Equatorial Guinea | 1,200.52 | 210.55 |  | 719.88 | 148.33 | 332.31 | 30.07 | 10.37 | 170.11 |
| Eritrea | 131.72 | 10.30 |  | 98.83 | 20.36 | 12.53 | 2.89 | 1.00 | 6.41 |
| Ethiopia | 182.85 | 10.54 |  | 139.79 | 28.80 | 14.25 | 2.41 | 0.83 | 7.29 |
| Fiji | 682.46 | 72.42 |  | 471.21 | 97.09 | 114.16 | 10.40 | 3.59 | 58.44 |
| Gabon | 582.83 | 117.73 |  | 325.52 | 67.07 | 190.24 | 15.12 | 5.22 | 97.39 |
| Gambia | 163.44 | 9.68 |  | 126.52 | 26.07 | 10.85 | 3.07 | 1.06 | 5.56 |
| Georgia | 702.56 | 60.21 |  | 510.84 | 105.26 | 86.46 | 11.86 | 4.09 | 44.26 |
| Ghana | 499.05 | 26.29 |  | 387.66 | 79.87 | 31.52 | 7.55 | 2.61 | 16.14 |
| Grenada | 1,304.46 | 167.84 |  | 905.82 | 186.64 | 212.00 | 44.10 | 15.21 | 108.53 |
| Guatemala | 822.26 | 66.90 |  | 607.30 | 125.13 | 89.83 | 15.55 | 5.36 | 45.99 |
| Guinea | 83.85 | 8.68 |  | 59.39 | 12.24 | 12.23 | 1.80 | 0.62 | 6.26 |
| Guinea-Bissau | 128.60 | 10.90 |  | 95.70 | 19.72 | 13.19 | 3.08 | 1.06 | 6.75 |
| Guyana | 742.59 | 62.98 |  | 536.96 | 110.64 | 94.99 | 10.68 | 3.68 | 48.62 |
| Haiti | 180.51 | 17.59 |  | 134.06 | 27.62 | 18.83 | 5.91 | 2.04 | 9.64 |
| Honduras | 621.81 | 46.93 |  | 467.32 | 96.29 | 58.20 | 12.75 | 4.40 | 29.79 |
| India | 189.78 | 23.63 |  | 126.96 | 26.16 | 36.67 | 3.61 | 1.25 | 18.77 |
| Indonesia | 865.21 | 54.47 |  | 653.54 | 134.66 | 77.02 | 11.19 | 3.86 | 39.42 |
| Iran, Islamic Rep. | 1,454.93 | 88.76 |  | 1,102.51 | 227.16 | 125.26 | 18.32 | 6.32 | 64.12 |
| Iraq | 601.87 | 88.46 |  | 404.71 | 83.39 | 113.77 | 22.46 | 7.75 | 58.24 |
| Jamaica | 1,683.00 | 113.47 |  | 1,298.05 | 267.45 | 117.50 | 39.64 | 13.67 | 60.15 |
| Jordan | 483.80 | 90.09 |  | 306.88 | 63.23 | 113.69 | 23.71 | 8.18 | 58.20 |
| Kazakhstan | 754.90 | 145.11 |  | 425.38 | 87.65 | 241.87 | 15.83 | 5.46 | 123.82 |
| Kenya | 338.69 | 24.17 |  | 254.56 | 52.45 | 31.68 | 5.91 | 2.04 | 16.22 |
| Kiribati | 461.01 | 28.01 |  | 355.07 | 73.16 | 32.78 | 8.35 | 2.88 | 16.78 |
| Korea, Dem. People's Rep. | 133.98 | 11.46 |  | 98.83 | 20.36 | 14.79 | 2.89 | 1.00 | 7.57 |
| Kosovo | 530.37 | 53.63 |  | 371.97 | 76.64 | 81.75 | 8.76 | 3.02 | 41.85 |
| Kyrgyzstan | 137.02 | 20.49 |  | 92.56 | 19.07 | 25.39 | 5.57 | 1.92 | 13.00 |
| Lao PDR | 613.76 | 29.34 |  | 474.20 | 97.71 | 41.85 | 5.89 | 2.03 | 21.42 |
| Lebanon | 2,213.74 | 161.60 |  | 1,681.98 | 346.56 | 185.21 | 49.66 | 17.13 | 94.81 |
| Lesotho | 278.57 | 27.06 |  | 210.62 | 43.40 | 24.56 | 10.77 | 3.72 | 12.57 |
| Liberia | 13.53 | 5.48 |  | 2.52 | 0.52 | 10.49 | 0.08 | 0.03 | 5.37 |
| Libya | 529.42 | 76.52 |  | 333.68 | 68.75 | 126.98 | 8.56 | 2.95 | 65.00 |
| Macedonia, FYR | 569.07 | 71.49 |  | 379.25 | 78.14 | 111.68 | 10.65 | 3.67 | 57.17 |
| Madagascar | 78.36 | 6.56 |  | 57.31 | 11.81 | 9.25 | 1.36 | 0.47 | 4.73 |
| Malawi | 131.25 | 9.07 |  | 101.73 | 20.96 | 8.56 | 3.48 | 1.20 | 4.38 |
| Malaysia | 2,883.40 | 140.15 |  | 2,204.39 | 454.20 | 224.81 | 18.64 | 6.43 | 115.08 |
| Maldives | 9,212.96 | 250.72 |  | 7,478.79 | 1,540.96 | 193.22 | 112.87 | 38.94 | 98.91 |
| Mali | 179.50 | 14.17 |  | 135.02 | 27.82 | 16.67 | 4.19 | 1.45 | 8.53 |
| Marshall Islands | 1,547.70 | 79.08 |  | 1,218.68 | 251.10 | 77.92 | 29.14 | 10.05 | 39.89 |
| Mauritania | 158.40 | 21.21 |  | 105.18 | 21.67 | 31.55 | 3.76 | 1.30 | 16.15 |
| Mauritius | 3,236.27 | 160.99 |  | 2,506.83 | 516.52 | 212.93 | 38.66 | 13.34 | 109.00 |
| Mexico | 1,773.48 | 170.95 |  | 1,298.66 | 267.58 | 207.24 | 48.23 | 16.64 | 106.09 |
| Micronesia, Fed. Sts. | 721.97 | 53.14 |  | 541.09 | 111.49 | 69.39 | 13.10 | 4.52 | 35.52 |
| Moldova | 243.15 | 29.83 |  | 166.34 | 34.27 | 42.53 | 5.99 | 2.07 | 21.77 |
| Mongolia | 283.73 | 56.00 |  | 159.54 | 32.87 | 91.31 | 6.88 | 2.37 | 46.74 |
| Montenegro | 701.49 | 91.61 |  | 459.36 | 94.65 | 147.48 | 11.98 | 4.13 | 75.50 |
| Morocco | 960.52 | 50.33 |  | 741.50 | 152.78 | 66.24 | 12.21 | 4.21 | 33.91 |
| Mozambique | 124.16 | 9.52 |  | 92.85 | 19.13 | 12.18 | 2.44 | 0.84 | 6.23 |
| Myanmar | 623.68 | 21.09 |  | 494.96 | 101.98 | 26.73 | 5.51 | 1.90 | 13.68 |
| Namibia | 992.86 | 122.78 |  | 734.05 | 151.25 | 107.56 | 50.35 | 17.37 | 55.06 |
| Nauru | 1,312.91 | 133.36 |  | 934.94 | 192.64 | 185.33 | 28.61 | 9.87 | 94.87 |
| Nepal | 116.72 | 13.68 |  | 82.60 | 17.02 | 17.11 | 3.66 | 1.26 | 8.76 |
| Nicaragua | 542.73 | 34.56 |  | 410.19 | 84.52 | 48.03 | 7.41 | 2.56 | 24.59 |
| Niger | 73.89 | 6.99 |  | 54.42 | 11.21 | 8.26 | 2.06 | 0.71 | 4.23 |
| Nigeria | 306.03 | 42.57 |  | 202.76 | 41.78 | 61.49 | 8.25 | 2.85 | 31.48 |
| Pakistan | 129.75 | 19.12 |  | 80.21 | 16.53 | 33.02 | 1.65 | 0.57 | 16.90 |
| Papua New Guinea | 272.52 | 33.73 |  | 182.68 | 37.64 | 52.20 | 5.21 | 1.80 | 26.72 |
| Paraguay | 996.24 | 82.73 |  | 748.17 | 154.16 | 93.92 | 25.77 | 8.89 | 48.08 |
| Peru | 926.11 | 94.66 |  | 652.88 | 134.52 | 138.71 | 17.59 | 6.07 | 71.00 |
| Philippines | 1,281.69 | 46.48 |  | 1,007.30 | 207.55 | 66.84 | 9.12 | 3.15 | 34.21 |
| Romania | 566.50 | 135.80 |  | 298.35 | 61.47 | 206.68 | 22.31 | 7.69 | 105.80 |
| Russian Federation | 695.91 | 126.25 |  | 399.00 | 82.21 | 214.70 | 12.15 | 4.19 | 109.91 |
| Rwanda | 362.71 | 12.02 |  | 287.43 | 59.22 | 16.05 | 2.83 | 0.98 | 8.22 |
| Samoa | 729.65 | 63.71 |  | 529.84 | 109.17 | 90.64 | 12.87 | 4.44 | 46.40 |
| São Tomé and Principe | 307.03 | 31.18 |  | 222.72 | 45.89 | 38.41 | 8.56 | 2.95 | 19.66 |
| Senegal | 185.94 | 15.26 |  | 137.01 | 28.23 | 20.70 | 3.47 | 1.20 | 10.60 |
| Serbia | 891.57 | 83.09 |  | 639.31 | 131.73 | 120.53 | 15.90 | 5.49 | 61.70 |
| Sierra Leone | 236.44 | 11.78 |  | 183.58 | 37.83 | 15.03 | 3.04 | 1.05 | 7.69 |
| Solomon Islands | 433.22 | 33.04 |  | 322.29 | 66.41 | 44.53 | 7.62 | 2.63 | 22.79 |
| Somalia | 131.83 | 10.36 |  | 98.83 | 20.36 | 12.64 | 2.89 | 1.00 | 6.47 |
| South Africa | 905.16 | 155.33 |  | 641.40 | 132.16 | 131.60 | 65.40 | 22.56 | 67.37 |
| South Sudan | 62.59 | 10.38 |  | 37.95 | 7.82 | 16.81 | 1.32 | 0.46 | 8.61 |
| Sri Lanka | 785.80 | 58.00 |  | 576.64 | 118.81 | 90.36 | 8.73 | 3.01 | 46.25 |
| St. Lucia | 1,423.48 | 142.07 |  | 1,032.67 | 212.78 | 178.03 | 37.87 | 13.06 | 91.14 |
| St. Vincent and the Grenadines | 884.47 | 119.35 |  | 604.77 | 124.61 | 155.09 | 29.71 | 10.25 | 79.39 |
| Sudan | 483.72 | 39.23 |  | 355.01 | 73.15 | 55.57 | 8.02 | 2.77 | 28.45 |
| Suriname | 1,352.03 | 171.52 |  | 940.04 | 193.69 | 218.29 | 44.44 | 15.33 | 111.74 |
| Swaziland | 1,312.33 | 76.21 |  | 1,027.06 | 211.62 | 73.65 | 28.63 | 9.88 | 37.70 |
| Syrian Arab Republic | 133.98 | 11.46 |  | 98.83 | 20.36 | 14.79 | 2.89 | 1.00 | 7.57 |
| Tajikistan | 108.86 | 14.57 |  | 72.60 | 14.96 | 21.31 | 2.72 | 0.94 | 10.91 |
| Tanzania | 112.55 | 14.49 |  | 76.55 | 15.77 | 20.23 | 3.08 | 1.06 | 10.36 |
| Thailand | 1,347.51 | 108.26 |  | 1,006.34 | 207.35 | 133.82 | 29.56 | 10.20 | 68.50 |
| Timor-Leste | 258.66 | 17.41 |  | 191.25 | 39.40 | 28.00 | 2.29 | 0.79 | 14.34 |
| Togo | 108.38 | 9.93 |  | 79.18 | 16.31 | 12.88 | 2.48 | 0.86 | 6.59 |
| Tonga | 590.12 | 62.16 |  | 411.10 | 84.70 | 94.32 | 10.32 | 3.56 | 48.28 |
| Tunisia | 829.29 | 62.53 |  | 614.67 | 126.65 | 87.97 | 13.01 | 4.49 | 45.03 |
| Turkey | 832.82 | 126.96 |  | 516.40 | 106.40 | 210.02 | 14.46 | 4.99 | 107.51 |
| Turkmenistan | 1,281.14 | 117.09 |  | 934.94 | 192.64 | 153.56 | 28.61 | 9.87 | 78.61 |
| Tuvalu | 1,203.41 | 77.30 |  | 934.94 | 192.64 | 75.83 | 28.61 | 9.87 | 38.82 |
| Uganda | 185.12 | 12.99 |  | 140.04 | 28.85 | 16.23 | 3.48 | 1.20 | 8.31 |
| Ukraine | 169.80 | 32.49 |  | 100.44 | 20.69 | 48.67 | 5.63 | 1.94 | 24.92 |
| Uzbekistan | 252.88 | 30.92 |  | 168.99 | 34.82 | 49.07 | 4.32 | 1.49 | 25.12 |
| Vanuatu | 455.40 | 44.98 |  | 324.07 | 66.77 | 64.56 | 8.87 | 3.06 | 33.05 |
| Venezuela, RB | 1,409.85 | 182.98 |  | 934.94 | 192.64 | 282.26 | 28.61 | 9.87 | 144.49 |
| Vietnam | 728.35 | 43.71 |  | 563.64 | 116.13 | 48.58 | 14.01 | 4.83 | 24.87 |
| West Bank and Gaza | 514.59 | 45.55 |  | 371.97 | 76.64 | 65.98 | 8.76 | 3.02 | 33.77 |
| Yemen, Rep. | 306.56 | 28.55 |  | 227.35 | 46.84 | 32.36 | 8.91 | 3.07 | 16.57 |
| Zambia | 231.68 | 26.82 |  | 167.20 | 34.45 | 30.03 | 8.51 | 2.94 | 15.37 |
| Zimbabwe | 140.46 | 14.78 |  | 98.83 | 20.36 | 21.27 | 2.89 | 1.00 | 10.89 |

***Notes:***

*Countries with missing unit cost estimates were replaced with the country income group averages.*

*Drug costs were excluded while estimating these unit costs as the IHME unit costs already included cost of drugs.*
