# Supplementary material for: Cost of illness for childhood diarrhea in low- and middle-income countries: a systematic review of evidence and modelled estimates
Source: BMC Public Health. 2020 May 5;20:619. doi: 10.1186/s12889-020-08595-8 (PMC7201538; doi:10.1186/s12889-020-08595-8)
Supplement: Supplementary file 5 — Additional file 5. Modelled cost of illness (2015 USD) estimates by country, excluding cost associated with oral rehydrated solution (ORS). [file 12889_2020_8595_MOESM5_ESM.docx]

**S5 Appendix: Modelled cost of illness (2015 USD) estimates by country, excluding cost associated with oral rehydration solution (ORS)**

| **Country** | **Inpatient** | **Outpatient** | **Inpatient-Direct Medical** | **Inpatient-Direct Non-Medical** | **Inpatient-Indirect** | **Outpatient-Direct Medical** | **Outpatient-Direct Non-Medical** | **Outpatient-Indirect** |
| --- | --- | --- | --- | --- | --- | --- | --- | --- |
| Afghanistan | 23.86 | 9.01 | 7.26 | 2.93 | 13.68 | 0.60 | 1.41 | 7.00 |
| Albania | 291.64 | 56.63 | 165.35 | 35.50 | 90.79 | 6.65 | 3.50 | 46.48 |
| Algeria | 244.63 | 56.92 | 122.38 | 26.65 | 95.60 | 5.05 | 2.94 | 48.94 |
| American Samoa | 592.12 | 151.06 | 269.53 | 56.97 | 265.62 | 10.33 | 4.76 | 135.97 |
| Angola | 305.35 | 60.38 | 173.73 | 37.23 | 94.39 | 8.08 | 3.99 | 48.32 |
| Armenia | 223.78 | 49.61 | 117.78 | 25.70 | 80.30 | 5.43 | 3.08 | 41.10 |
| Azerbaijan | 333.07 | 74.81 | 170.08 | 36.48 | 126.52 | 6.58 | 3.47 | 64.76 |
| Bangladesh | 58.24 | 17.63 | 23.98 | 6.37 | 27.89 | 1.60 | 1.75 | 14.27 |
| Belarus | 445.13 | 81.02 | 258.09 | 54.61 | 132.43 | 8.94 | 4.29 | 67.79 |
| Belize | 380.13 | 71.29 | 220.90 | 46.95 | 112.28 | 9.38 | 4.44 | 57.48 |
| Benin | 46.11 | 12.25 | 22.50 | 6.07 | 17.54 | 1.54 | 1.73 | 8.98 |
| Bhutan | 168.56 | 37.96 | 87.89 | 19.54 | 61.12 | 4.07 | 2.60 | 31.29 |
| Bolivia | 193.63 | 44.08 | 100.65 | 22.17 | 70.81 | 4.93 | 2.90 | 36.25 |
| Bosnia and Herzegovina | 323.82 | 61.42 | 186.22 | 39.80 | 97.79 | 7.55 | 3.81 | 50.06 |
| Botswana | 602.89 | 93.51 | 377.33 | 79.18 | 146.37 | 12.92 | 5.66 | 74.93 |
| Brazil | 285.99 | 106.37 | 70.35 | 15.93 | 199.71 | 2.18 | 1.95 | 102.23 |
| Bulgaria | 536.37 | 98.04 | 310.10 | 65.33 | 160.95 | 10.75 | 4.91 | 82.39 |
| Burkina Faso | 35.59 | 9.78 | 17.06 | 4.95 | 13.57 | 1.21 | 1.62 | 6.95 |
| Burundi | 16.81 | 5.33 | 7.46 | 2.97 | 6.38 | 0.64 | 1.42 | 3.26 |
| Cabo Verde | 177.68 | 43.57 | 87.36 | 19.43 | 70.89 | 4.53 | 2.76 | 36.29 |
| Cambodia | 64.74 | 17.37 | 30.38 | 7.69 | 26.67 | 1.87 | 1.85 | 13.65 |
| Cameroon | 71.77 | 18.46 | 35.09 | 8.66 | 28.01 | 2.17 | 1.95 | 14.34 |
| Central African Republic | 27.03 | 6.67 | 15.06 | 4.54 | 7.44 | 1.24 | 1.63 | 3.81 |
| Chad | 43.67 | 12.22 | 20.22 | 5.60 | 17.85 | 1.40 | 1.68 | 9.14 |
| China | 446.97 | 108.59 | 215.44 | 45.82 | 185.70 | 9.17 | 4.36 | 95.06 |
| Colombia | 412.46 | 94.88 | 225.24 | 47.84 | 139.37 | 16.60 | 6.93 | 71.35 |
| Comoros | 43.65 | 11.77 | 21.31 | 5.82 | 16.51 | 1.58 | 1.74 | 8.45 |
| DRC | 21.15 | 7.57 | 7.64 | 3.01 | 10.50 | 0.74 | 1.46 | 5.37 |
| Congo, Rep. | 113.20 | 26.91 | 57.34 | 13.25 | 42.60 | 2.90 | 2.20 | 21.81 |
| Costa Rica | 837.08 | 157.52 | 478.01 | 99.93 | 259.14 | 17.59 | 7.27 | 132.65 |
| Côte d'Ivoire | 73.13 | 20.49 | 32.75 | 8.18 | 32.20 | 2.09 | 1.92 | 16.48 |
| Cuba | 511.88 | 110.86 | 277.26 | 58.56 | 176.06 | 14.52 | 6.21 | 90.13 |
| Djibouti | 105.92 | 28.15 | 49.52 | 11.64 | 44.76 | 3.00 | 2.24 | 22.92 |
| Dominica | 757.45 | 108.93 | 491.06 | 102.61 | 163.77 | 17.76 | 7.33 | 83.84 |
| Dominican Republic | 428.18 | 89.66 | 230.41 | 48.91 | 148.86 | 9.11 | 4.34 | 76.20 |
| Ecuador | 469.30 | 88.19 | 269.53 | 56.97 | 142.80 | 10.33 | 4.76 | 73.10 |
| Egypt, Arab Rep. | 204.47 | 49.77 | 99.37 | 21.91 | 83.19 | 4.45 | 2.74 | 42.58 |
| El Salvador | 123.00 | 52.05 | 20.29 | 5.61 | 97.10 | 0.85 | 1.49 | 49.71 |
| Equatorial Guinea | 1,218.40 | 198.43 | 733.52 | 152.57 | 332.31 | 20.16 | 8.16 | 170.11 |
| Eritrea | 22.03 | 8.39 | 6.69 | 2.81 | 12.53 | 0.57 | 1.40 | 6.41 |
| Ethiopia | 33.26 | 9.99 | 14.58 | 4.44 | 14.25 | 1.11 | 1.58 | 7.29 |
| Fiji | 272.99 | 68.32 | 130.51 | 28.32 | 114.16 | 6.45 | 3.43 | 58.44 |
| Gabon | 605.30 | 114.46 | 342.96 | 72.10 | 190.24 | 11.80 | 5.27 | 97.39 |
| Gambia | 53.91 | 9.72 | 34.52 | 8.55 | 10.85 | 2.20 | 1.96 | 5.56 |
| Georgia | 205.95 | 51.74 | 97.88 | 21.60 | 86.46 | 4.66 | 2.81 | 44.26 |
| Ghana | 68.70 | 20.01 | 29.64 | 7.54 | 31.52 | 1.99 | 1.89 | 16.14 |
| Grenada | 694.29 | 130.15 | 398.70 | 83.58 | 212.00 | 15.19 | 6.44 | 108.53 |
| Guatemala | 231.98 | 54.85 | 116.67 | 25.47 | 89.83 | 5.69 | 3.16 | 45.99 |
| Guinea | 29.16 | 8.76 | 12.85 | 4.08 | 12.23 | 0.97 | 1.53 | 6.26 |
| Guinea-Bissau | 35.81 | 9.68 | 17.57 | 5.05 | 13.19 | 1.29 | 1.64 | 6.75 |
| Guyana | 255.13 | 59.91 | 131.59 | 28.55 | 94.99 | 7.50 | 3.79 | 48.62 |
| Haiti | 50.07 | 13.36 | 24.72 | 6.53 | 18.83 | 1.87 | 1.85 | 9.64 |
| Honduras | 168.06 | 37.33 | 89.90 | 19.96 | 58.20 | 4.71 | 2.82 | 29.79 |
| India | 89.85 | 23.05 | 42.91 | 10.28 | 36.67 | 2.29 | 1.99 | 18.77 |
| Indonesia | 152.72 | 44.77 | 61.59 | 14.12 | 77.02 | 3.08 | 2.26 | 39.42 |
| Iran, Islamic Rep. | 428.99 | 76.69 | 250.65 | 53.08 | 125.26 | 8.45 | 4.12 | 64.12 |
| Iraq | 182.49 | 63.37 | 55.79 | 12.93 | 113.77 | 2.92 | 2.21 | 58.24 |
| Jamaica | 386.67 | 74.04 | 222.00 | 47.17 | 117.50 | 9.43 | 4.45 | 60.15 |
| Jordan | 234.37 | 65.52 | 98.87 | 21.81 | 113.69 | 4.55 | 2.77 | 58.20 |
| Kazakhstan | 614.96 | 139.91 | 308.16 | 64.93 | 241.87 | 11.07 | 5.02 | 123.82 |
| Kenya | 67.98 | 20.03 | 28.90 | 7.39 | 31.68 | 1.94 | 1.87 | 16.22 |
| Kiribati | 123.65 | 24.10 | 74.16 | 16.71 | 32.78 | 4.55 | 2.77 | 16.78 |
| Korea, Dem. People's Rep. | 38.79 | 10.54 | 18.71 | 5.29 | 14.79 | 1.32 | 1.65 | 7.57 |
| Kosovo | 164.22 | 47.78 | 67.19 | 15.28 | 81.75 | 3.52 | 2.41 | 41.85 |
| Kyrgyzstan | 65.55 | 16.81 | 32.11 | 8.05 | 25.39 | 1.94 | 1.87 | 13.00 |
| Lao PDR | 89.28 | 25.66 | 38.14 | 9.29 | 41.85 | 2.26 | 1.98 | 21.42 |
| Lebanon | 769.19 | 118.69 | 483.03 | 100.96 | 185.21 | 16.86 | 7.02 | 94.81 |
| Lesotho | 60.97 | 16.33 | 29.01 | 7.41 | 24.56 | 1.90 | 1.86 | 12.57 |
| Liberia | 22.79 | 7.64 | 9.01 | 3.29 | 10.49 | 0.79 | 1.47 | 5.37 |
| Libya | 492.29 | 79.29 | 301.71 | 63.60 | 126.98 | 9.73 | 4.56 | 65.00 |
| Macedonia, FYR | 371.73 | 69.11 | 214.43 | 45.62 | 111.68 | 7.98 | 3.95 | 57.17 |
| Madagascar | 22.73 | 6.94 | 9.99 | 3.49 | 9.25 | 0.75 | 1.46 | 4.73 |
| Malawi | 23.57 | 6.76 | 11.26 | 3.75 | 8.56 | 0.88 | 1.50 | 4.38 |
| Malaysia | 626.30 | 131.27 | 331.71 | 69.78 | 224.81 | 11.15 | 5.05 | 115.08 |
| Maldives | 544.34 | 116.19 | 289.94 | 61.17 | 193.22 | 11.96 | 5.33 | 98.91 |
| Mali | 40.59 | 11.55 | 18.65 | 5.28 | 16.67 | 1.35 | 1.67 | 8.53 |
| Marshall Islands | 188.82 | 48.47 | 90.76 | 20.13 | 77.92 | 5.49 | 3.09 | 39.89 |
| Mauritania | 68.06 | 19.73 | 29.08 | 7.43 | 31.55 | 1.77 | 1.81 | 16.15 |
| Mauritius | 699.87 | 128.64 | 402.56 | 84.38 | 212.93 | 13.71 | 5.93 | 109.00 |
| Mexico | 819.72 | 129.94 | 506.65 | 105.83 | 207.24 | 16.85 | 7.01 | 106.09 |
| Micronesia, Fed. Sts. | 235.12 | 46.61 | 136.23 | 29.50 | 69.39 | 7.35 | 3.74 | 35.52 |
| Moldova | 106.50 | 26.82 | 51.85 | 12.12 | 42.53 | 2.86 | 2.19 | 21.77 |
| Mongolia | 170.24 | 52.35 | 64.26 | 14.67 | 91.31 | 3.28 | 2.33 | 46.74 |
| Montenegro | 524.41 | 91.45 | 311.35 | 65.58 | 147.48 | 10.97 | 4.98 | 75.50 |
| Morocco | 205.16 | 42.57 | 114.00 | 24.92 | 66.24 | 5.55 | 3.12 | 33.91 |
| Mozambique | 30.81 | 8.94 | 14.26 | 4.37 | 12.18 | 1.12 | 1.59 | 6.23 |
| Myanmar | 29.64 | 14.90 | 1.22 | 1.69 | 26.73 | 0.01 | 1.20 | 13.68 |
| Namibia | 296.81 | 65.42 | 155.73 | 33.52 | 107.56 | 6.81 | 3.55 | 55.06 |
| Nauru | 262.33 | 101.25 | 62.66 | 14.34 | 185.33 | 3.85 | 2.53 | 94.87 |
| Nepal | 37.68 | 11.44 | 15.87 | 4.70 | 17.11 | 1.10 | 1.58 | 8.76 |
| Nicaragua | 129.34 | 30.51 | 66.23 | 15.08 | 48.03 | 3.51 | 2.41 | 24.59 |
| Niger | 20.14 | 6.40 | 8.66 | 3.22 | 8.26 | 0.72 | 1.45 | 4.23 |
| Nigeria | 117.78 | 36.39 | 45.49 | 10.81 | 61.49 | 2.76 | 2.15 | 31.48 |
| Pakistan | 74.44 | 20.68 | 33.16 | 8.27 | 33.02 | 1.91 | 1.86 | 16.90 |
| Papua New Guinea | 140.61 | 33.77 | 72.12 | 16.29 | 52.20 | 4.35 | 2.70 | 26.72 |
| Paraguay | 257.58 | 57.53 | 134.51 | 29.15 | 93.92 | 6.14 | 3.32 | 48.08 |
| Peru | 445.23 | 85.57 | 252.97 | 53.56 | 138.71 | 9.94 | 4.63 | 71.00 |
| Philippines | 155.81 | 40.46 | 72.58 | 16.39 | 66.84 | 3.75 | 2.49 | 34.21 |
| Romania | 662.04 | 124.19 | 376.38 | 78.98 | 206.68 | 12.78 | 5.61 | 105.80 |
| Russian Federation | 833.37 | 132.12 | 511.78 | 106.88 | 214.70 | 15.62 | 6.59 | 109.91 |
| Rwanda | 38.56 | 11.13 | 17.47 | 5.03 | 16.05 | 1.28 | 1.64 | 8.22 |
| Samoa | 252.52 | 56.68 | 133.03 | 28.84 | 90.64 | 6.75 | 3.53 | 46.40 |
| São Tomé and Principe | 85.44 | 24.18 | 37.80 | 9.22 | 38.41 | 2.47 | 2.05 | 19.66 |
| Senegal | 57.19 | 14.33 | 29.06 | 7.42 | 20.70 | 1.88 | 1.85 | 10.60 |
| Serbia | 406.03 | 74.73 | 235.54 | 49.96 | 120.53 | 8.79 | 4.23 | 61.70 |
| Sierra Leone | 37.86 | 10.67 | 17.74 | 5.09 | 15.03 | 1.32 | 1.66 | 7.69 |
| Solomon Islands | 176.44 | 32.48 | 108.19 | 23.73 | 44.53 | 6.31 | 3.38 | 22.79 |
| Somalia | 36.64 | 9.44 | 18.71 | 5.29 | 12.64 | 1.32 | 1.65 | 6.47 |
| South Africa | 531.58 | 85.57 | 330.46 | 69.52 | 131.60 | 12.64 | 5.56 | 67.37 |
| South Sudan | 40.81 | 11.58 | 18.71 | 5.29 | 16.81 | 1.32 | 1.65 | 8.61 |
| Sri Lanka | 186.11 | 52.46 | 78.21 | 17.55 | 90.36 | 3.72 | 2.48 | 46.25 |
| St. Lucia | 721.75 | 114.94 | 449.64 | 94.08 | 178.03 | 16.80 | 7.00 | 91.14 |
| St. Vincent and the Grenadines | 591.13 | 99.05 | 360.36 | 75.68 | 155.09 | 13.72 | 5.93 | 79.39 |
| Sudan | 127.81 | 34.35 | 58.71 | 13.53 | 55.57 | 3.50 | 2.41 | 28.45 |
| Suriname | 501.30 | 126.02 | 233.47 | 49.54 | 218.29 | 9.72 | 4.55 | 111.74 |
| Swaziland | 188.81 | 44.89 | 94.30 | 20.86 | 73.65 | 4.46 | 2.74 | 37.70 |
| Syrian Arab Republic | 25.83 | 9.20 | 7.96 | 3.07 | 14.79 | 0.32 | 1.31 | 7.57 |
| Tajikistan | 54.92 | 14.38 | 26.68 | 6.93 | 21.31 | 1.69 | 1.78 | 10.91 |
| Tanzania | 44.89 | 13.36 | 19.26 | 5.40 | 20.23 | 1.34 | 1.66 | 10.36 |
| Thailand | 342.53 | 78.95 | 171.87 | 36.85 | 133.82 | 6.88 | 3.57 | 68.50 |
| Timor-Leste | 53.19 | 17.32 | 19.70 | 5.49 | 28.00 | 1.33 | 1.66 | 14.34 |
| Togo | 29.51 | 9.08 | 12.60 | 4.03 | 12.88 | 0.96 | 1.53 | 6.59 |
| Tonga | 276.28 | 59.47 | 149.68 | 32.27 | 94.32 | 7.42 | 3.76 | 48.28 |
| Tunisia | 301.68 | 55.50 | 176.01 | 37.70 | 87.97 | 6.89 | 3.58 | 45.03 |
| Turkey | 737.72 | 128.20 | 436.36 | 91.34 | 210.02 | 14.49 | 6.20 | 107.51 |
| Turkmenistan | 389.03 | 90.55 | 194.06 | 41.42 | 153.56 | 7.99 | 3.96 | 78.61 |
| Tuvalu | 221.35 | 49.47 | 119.47 | 26.05 | 75.83 | 7.03 | 3.62 | 38.82 |
| Uganda | 37.47 | 11.08 | 16.42 | 4.82 | 16.23 | 1.17 | 1.60 | 8.31 |
| Ukraine | 160.89 | 31.51 | 91.86 | 20.36 | 48.67 | 4.01 | 2.58 | 24.92 |
| Uzbekistan | 149.29 | 32.46 | 81.91 | 18.31 | 49.07 | 4.57 | 2.78 | 25.12 |
| Vanuatu | 305.10 | 47.63 | 198.26 | 42.28 | 64.56 | 9.95 | 4.63 | 33.05 |
| Venezuela, RB | 1,018.28 | 175.46 | 609.09 | 126.93 | 282.26 | 22.13 | 8.83 | 144.49 |
| Vietnam | 109.13 | 29.72 | 49.02 | 11.53 | 48.58 | 2.71 | 2.14 | 24.87 |
| West Bank and Gaza | 148.44 | 39.70 | 67.19 | 15.28 | 65.98 | 3.52 | 2.41 | 33.77 |
| Yemen, Rep. | 150.75 | 25.61 | 96.97 | 21.41 | 32.36 | 5.83 | 3.21 | 16.57 |
| Zambia | 56.56 | 18.47 | 20.81 | 5.72 | 30.03 | 1.41 | 1.69 | 15.37 |
| Zimbabwe | 45.27 | 13.86 | 18.71 | 5.29 | 21.27 | 1.32 | 1.65 | 10.89 |
