# Supplementary material for: Cost of illness for childhood diarrhea in low- and middle-income countries: a systematic review of evidence and modelled estimates
Source: BMC Public Health. 2020 May 5;20:619. doi: 10.1186/s12889-020-08595-8 (PMC7201538; doi:10.1186/s12889-020-08595-8)
Supplement: Supplementary file 6 — Additional file 6. Comparison of modelled and empirical estimates of direct medical costs across countries. [file 12889_2020_8595_MOESM6_ESM.docx]

**S6 Appendix: Comparison of modelled and empirical estimates of direct medical costs across countries**

|  |
| --- |
|  |

Notes: Please use the secondary axis to read values in green dots. For the others, please use the primary axis.

- The yellow dots represent empirical estimates from the literature
- The blue bars represent modelled estimates using the WHO CHOICE service delivery unit costs as inputs
- The green dots represent modelled estimates using the IHME service delivery unit costs as inputs
